# Enhancing gemcitabine uptake by promoting dimerization of ENT1 transporter improves response rates of patients with sarcoma

**DOI:** 10.1172/JCI183474

**Published:** 2026-09-03

**Authors:** Aditya Ganju, Shyam Rao, Mark A. Dickson, Robert A. Lefkowitz, Chris Thompson, Jin Cheng, Katia Manova, Adriana Haimovitz-Friedman, Gary Schwartz, Zhigang Zhang, Zvi Fuks, William D. Tap, Richard Kolesnick

**Affiliations:** 1 Laboratory of Signal Transduction,; 2 Department of Radiation Oncology,; 3 Department of Medicine,; 4 Department of Radiology,; 5 Molecular Cytology Core Facility, and; 6 Department of Epidemiology and Biostatistics, Memorial Sloan Kettering Cancer Center, New York, New York, USA.

**Keywords:** Cell biology, Oncology, Cancer, Molecular biology, Transport

## Abstract

Transport of nucleoside chemotherapeutic drugs into tumor cells is primarily accomplished through equilibrative nucleoside transporter 1 (ENT1), which is considered constitutively active. Drugs are redistributed via ENT1 across lipid bilayers via facilitated diffusion. Here we discover that ENT1 is not constitutively active, but rather requires activation of acid sphingomyelinase (ASMase) by gemcitabine, generating ceramide-rich platforms (CRPs) on external plasma membranes of endothelial and tumor cells into which ENT1 inserts, dimerizing therein to functionalize transmembrane gemcitabine transport. Whereas sarcoma cells synthesize minimal ASMase, they take up gemcitabine poorly in vitro and in murine xenografts. A strategy designed to augment gemcitabine-induced ASMase secretion into the extravascular space by ASMase-rich neo-angiogenic cells, which then targets tumor cell plasma membranes, yields “bystander” CRPs on sarcoma cells and ENT1 insertion therein, conferring markedly enhanced gemcitabine uptake and xenograft response. Engaging this biology in a prospective phase II clinical trial in advanced sarcoma yielded robust volumetric changes in evaluated tumors that developed early and were often durable.

## Abstract 2

ENT1 dimerizes in ceramide-rich platforms to functionalize gemcitabine uptake into fibrosarcoma cells. Enhancing this biology in a Phase II Advanced Sarcoma trial improved response rates.<br>

## Introduction

Sphingomyelin (SM) is a phospholipid preferentially concentrated in the outer plasma membrane of all mammalian cells, where it interacts with cholesterol and undergoes phase separation to form liquid-ordered microdomains (i.e., rafts). Diverse stresses (e.g., radiation, UVC rays, heat shock, TNF superfamily members, select chemotherapeutics) affect plasma membranes to induce, within seconds, translocation of the SM phosphodiesterase acid sphingomyelinase (ASMase) from secretory lysosomes that abut the inner plasma membrane to the SM-rich outer membrane (1, 2). Resultant ceramide generated therein coalesces into ceramide-rich platforms (CRPs), 0.5–2.0 μm macrodomains that form transiently on the cell surface, into which multisubunit proteins insert and oligomerize for the purpose of functionalizing transmembrane signaling (3). Endothelial cells are uniquely sensitive to ceramide-mediated signaling; they secrete up to 20-fold more ASMase than other mammalian cells (4), including all solid tumor cells studied to date. An expanding literature now indicates that an emerging form of radiation therapy, termed single-dose radiotherapy, which yields significantly higher local cure rates than conventional fractionated/hypofractionated radiotherapy, does so based on a synthetic lethality associated with ASMase/CRP-mediated vascular dysfunction enhancement of tumor-cell DNA damage unrepair (a “bystander effect”) (5, 6).

Recently we began investigating whether the genotoxic chemotherapeutic gemcitabine engages ASMase/ceramide signaling. We showed that only dividing endothelial cells, such as might be found in tumors, secrete ASMase in response to gemcitabine and undergo gemcitabine-induced apoptosis, because growth-arrested endothelial cells are resistant to 5 logs of gemcitabine, a state reversible by exogenous SMase (7). Furthermore, we showed VEGF is the principal inhibitor of ASMase secretion in murine sarcoma xenografts and that provision of an antiangiogenic agent to enhance ASMase secretion has a strict time window of 1 hour preceding chemotherapy to sensitize gemcitabine-induced tumor response (8). In the present study, we define the mechanism of tumor chemosensitization.

In normal cells, nucleosides are taken up by 2 families of transporter proteins: low-affinity equilibrative nucleoside transporters 1–4 (ENT1–4), of which ENT1 and ENT2 are widely distributed, and higher-affinity concentrative nucleoside transporters 1–3 (CNT1–3), which are more restricted in distribution (9–13). Evidence indicates that CNTs are often downregulated in cancer (14, 15), rendering nucleoside uptake preferentially ENT dependent. Consistent with this observation, ENT1 is considered the primary transporter of gemcitabine in many cancers (15–17) and, in select cancers, has been reported to be a favorable predictive (18–26) or prognostic marker of response to gemcitabine therapy (27, 28) (for an opposing result, see ref. 29). Translational data have been mixed, with 2 retrospective studies showing favorable correlation with outcome in pancreatic adenocarcinoma (20, 30), whereas a prospective study did not (31). Relative to the present report, a retrospective analysis of ENT expression by PCR of samples from 71 patients with advanced leiomyosarcoma or angiosarcoma revealed that patients with high ENT1 levels had favorable progression-free and overall survival (32).

Here we show that gemcitabine, although capable of exiting the microvasculature, does not efficiently cross a sarcoma cell membrane, because ENT1 must first insert into a CRP and dimerize therein to functionalize transport. Also, as sarcoma cells synthesize or secrete minimal ASMase, they fail to form CRPs. However, a 1-hour pretreatment with an antiangiogenic drug enhances microvascular, gemcitabine-induced endothelial cell ASMase secretion 3- to 5-fold, conferring bystander CRP formation onto sarcoma cells, markedly enhancing ENT1-mediated gemcitabine uptake in vitro and in sarcoma xenografts, yielding tumor cure. Furthermore, engaging this biology in a prospective phase II clinical trial in advanced sarcoma revealed encouraging volumetric changes in evaluated tumors that developed early and were often durable, supporting future clinical trial exploration.

## Results

### Gemcitabine-induced CRP formation is required for ENT1 functionalization of gemcitabine uptake.

Prior studies showed that gemcitabine induces ASMase activation within seconds to minutes in proliferative gemcitabine-sensitive, but not in growth-arrested, gemcitabine-resistant, bovine aortic endothelial cells (BAECs), a population that mimics the human and murine microvascular response to anticancer agents (33), conferring ceramide-mediated apoptotic death (7). Here we show that proliferative, but not growth-arrested, BAECs take up [^14^C]gemcitabine (10 μM gemcitabine plus tracer [^14^C]gemcitabine) under Michaelis-Menten kinetic conditions (*K_m_* = 1.5 μM) (Supplemental Figure 1A; supplemental material available online with this article; https://doi.org/10.1172/JCI183474DS1), a prerequisite for gemcitabine-induced apoptosis initiated by masked chain termination (34–36). Gemcitabine uptake occurs via ENT1, as dilazep and 6-[(4-nitrobenzyl) thio]-9-β-d-ribofuranosylpurine (NBMPR), highly specific ENT1 inhibitors, block [^14^C]gemcitabine uptake (Figure 1A). Note that gemcitabine-sensitive proliferative and gemcitabine-resistant growth-arrested BAEC display identical ENT1 protein and mRNA levels (Supplemental Figure 1B). These populations, however, differ in their capacity for gemcitabine-induced CRP formation. Using our previously published 2D assay for assessing CRP incidence (3, 37), we show untreated proliferative BAECs display minimal CRPs on their cell surface, with ENT1 distributed in a punctate fashion throughout (Figure 1B). At 1 minute after gemcitabine treatment, large CRPs and ENT1 aggregates are detected and colocalize by confocal microscopy. These events are detectable by 15 seconds of gemcitabine treatment, peak at 60 seconds, and dissipate by 5 minutes (Figure 1C). As little as 0.3 μM gemcitabine is effective, and a maximal effect occurs with 10 μM gemcitabine (Figure 1D). These events are absent in gemcitabine-resistant, growth-arrested BAECs, as we describe next.

To more fully appreciate CRP size, structure, and location, we developed a new volumetric detection system using confocal imaging and Imaris software to generate a 3D “movie,” which has the advantage of having a 2-log dynamic range. Stills from such movies (Figure 1E) show minimal ceramide on the surface of unstimulated proliferative BAECs compared with 20-fold larger CRPs at 1 minute after gemcitabine treatment (quantified in Figure 1F, full movies are shown in Supplemental Videos 1 and 2). Gemcitabine-resistant, growth-arrested BAECs do not activate ASMase after gemcitabine treatment (7), and they consequently fail to generate CRPs or redistribute ENT1 (Figure 1G), which is consistent with minimal gemcitabine uptake (Figure 1A) and lack of apoptosis (Supplemental Figure 1C), confirming apoptosis data published by van Hell et al. (7). Exposure of gemcitabine-resistant, growth-arrested BAECs to exogenous *Bacillus cereus* SMase (bSMase) to hydrolyze cell surface SM to ceramide, an established approach in the field (7, 38), pharmacologically generates CRPs (Figure 1G) without ENT1 insertion, gemcitabine uptake (Figure 1H), or apoptosis induction (Supplemental Figure 1C). Concurrent provision of bSMase and gemcitabine, however, confers the full proliferative BAEC phenotype onto growth-arrested BAECs with bSMase-induced CRP formation coupled with gemcitabine-induced ENT1 insertion (Figure 1G), quantified by 3D volumetric analysis as 8-fold above baseline (Supplemental Figure 1D), yielding gemcitabine uptake (Figure 1H), and apoptotic death (Supplemental Figure 1C; ref. 7). These data are consistent with gemcitabine uptake being a 2-step process involving membrane perturbation that permits ENT1 insertion into newly formed CRPs, functionalizing drug uptake.

### ASMase is obligate for gemcitabine action.

Anti-ceramide 6B5 single-chain variable fragment (scFv) pretreatment of proliferative BAECs, by binding monomeric ceramide generated on the cell surface, a strategy we recently published (39–41), abolishes gemcitabine-induced CRP formation and ENT1 redistribution (Figure 2A), preventing gemcitabine uptake (Figure 2B) and apoptosis induction (Figure 2C). Similarly, imipramine, which displaces ASMase from its lipid binding site on the inner lysosomal membrane, leading to its proteolytic degradation (42) and inhibiting gemcitabine-induced ASMase activation in proliferative BAECs (7, 43) (Supplemental Figure 2A), abrogates gemcitabine-induced CRP formation and ENT1 insertion therein (Figure 2D), gemcitabine uptake (Figure 2E), and gemcitabine-induced apoptosis (Figure 2F). These data further support gemcitabine-induced CRPs as mediating gemcitabine-induced cell death.

Because SM and cholesterol interact tightly on the mammalian cell surface, resulting in formation of lipid rafts (44), we tested whether altering raft structure might affect ENT1 functionalization. Depleting raft cholesterol to alter raft structure with methyl-β-cyclodextrin (45), a well-described technique, prevents gemcitabine-induced CRP formation in proliferative BAECs (Supplemental Figure 2B), suppressing ENT1-mediated gemcitabine uptake (Supplemental Figure 2C) and consequent apoptosis (Supplemental Figure 2D). Similarly, VEGF-165, the principal inhibitor of ASMase signaling in the hypoxic tumor microenvironment (46), blocks gemcitabine-induced ASMase activation (Figure 2G), CRP formation, and ENT1 colocalization (Figure 2H), suppressing gemcitabine uptake into proliferative BAECs (Figure 2I) and so inhibiting gemcitabine-induced cell death (Figure 2J). Together, these data provide a body of work supporting ASMase signaling as requisite for ENT1 functionalization of gemcitabine uptake in proliferating endothelial cells, which are prevalent in the tumor microenvironment (47).

Whereas nucleoside uptake is fundamental to eucaryotic RNA and DNA synthesis and crucial for nucleoside analog chemotherapeutic (e.g., gemcitabine, cytosine arabinoside, 6-mercaptopurine, azacytidine) efficacy, substantive gaps exist in understanding the mechanism of ENT-mediated nucleoside transport. In 2019, Grañe-Boladeras et al. (48) provided the first evidence that ENT1 homodimerizes or oligomerizes under conditions of active nucleoside transport, reminiscent of the active trimerization state of the CNT family (10). To investigate whether ENT1 multimerizes in a CRP, we used the Promega NanoLuc Binary Technology (NanoBiT) system to quantify protein-protein interaction among human ENTs (hENTs) in live cells (Figure 3A). This technology is composed of 2 small subunits — large BiT and small BiT — that are fused to proteins of interest. Once target proteins interact within a cell, the subunits come together and form an active enzyme that generates a bright luminescent signal. In ENT-null human embryonic kidney 293 (HEK293) cells expressing ENT1 fused to both large and small BiT subunits, gemcitabine increases luminescence within 30 seconds, an event that peaks at 60 seconds and dissipates thereafter (Figure 3B), indicative of transient dimerization coincident with kinetics of CRP formation and resolution (Figure 1C). Furthermore, anti-ceramide scFv not only prevents CRP formation, thereby abrogating ENT1-mediated gemcitabine uptake (Figure 2, A and B), it abolishes ENT1 dimerization (Figure 3C), consistent with dimerization or oligomerization as mediating ENT1 functionalization within a CRP. Complementary studies using HEK293 cells expressing ENT1–large BiT and ENT2–small BiT subunits show gemcitabine concomitantly reduces ENT1-ENT2 heterodimers (Figure 3D), a state associated with nucleobase uptake (48), indicating specificity of the gemcitabine-induced ENT1-ENT1 interaction within a CRP. Based on this evidence, we posit that gemcitabine activates its own transport by generating a membrane microenvironment that supports ENT1 insertion for the purpose of self-association, leading to functionalization.

### MCA/129 fibrosarcoma cells lacking ASMase fail to transport gemcitabine, which is reversible by exogenous SMase.

Whether ASMase signaling mediates CRP formation and ENT1 functionalization in tumor cells was investigated using the murine MCA/129 fibrosarcoma model, because favorable outcome of treatment with gemcitabine, a potential first-line therapy for many sarcoma subtypes, was recently associated with high ENT1 levels (32). We show that proliferative endothelial cells had significantly higher baseline ASMase activity compared with proliferative fibrosarcoma cells (Figure 4A), and only endothelial cells secrete detectable ASMase activity upon gemcitabine treatment (Figure 4B). Furthermore, proliferative MCA/129 cells had 10-fold less gemcitabine uptake compared with proliferative endothelial cells (Figure 4C), despite a comparable *K_m_* (2.5 μM) (Supplemental Figure 3, A and B) and ENT1 expression (Supplemental Figure 3C). Whereas gemcitabine-treated proliferative MCA/129 cells did not generate CRPs (Figure 4D), as with growth-arrested BAECs, exogenous bSMase induced dose-dependent CRP formation on their surface (Figure 4D and Supplemental Figure 4B), into which ENT1 inserted upon gemcitabine treatment, conferring markedly enhanced gemcitabine uptake (Figure 4E) and apoptosis (Figure 4F and Supplemental Figure 4A).

These studies indicate that MCA/129 fibrosarcoma cells contain minimal ASMase, an amount insufficient upon contact with gemcitabine to generate CRPs for ENT1 functionalization. However, external SMase rectifies this CRP generation defect, conferring ENT1-mediated gemcitabine transport, leading to sarcoma cell death.

### ASMase secreted by gemcitabine-treated endothelium confers a bystander tumor response.

Because exogenous ASMase permits gemcitabine-induced ENT1 activation leading to gemcitabine uptake, we investigated whether ASMase secreted by endothelial cells might similarly confer bystander CRP formation onto tumor cells, rendering ENT1-mediated gemcitabine uptake. For these studies, we deconstructed the potential interaction between endothelial cells and tumor cells in vitro into 2 phases. In the first phase, we isolated ASMase-containing medium from gemcitabine-treated endothelial cells and removed residual gemcitabine by size-exclusion chromatography. In the second phase, we incubated CRP-defective MCA/129 fibrosarcoma cells with this ASMase-containing conditioned medium and examined ENT1-mediated gemcitabine uptake by sarcoma cells (Figure 5A). This strategy confirmed that incubating sarcoma cells in ASMase-containing conditioned medium alone yielded bystander CRPs that lack ENT1 (Figure 5, B and C). Concomitant gemcitabine provision, however, conferred rapid ENT1 transit into CRPs (Figure 5C), followed by increased sarcoma cell gemcitabine uptake (Supplemental Figure 5C) and apoptosis (Supplemental Figure 5, A and B). To confirm that ASMase in conditioned medium mediates gemcitabine uptake and apoptosis, BAECs were pretreated with imipramine in the first phase of our assay to suppress ASMase secretion (Supplemental Figure 5D), a strategy successful in abrogating conditioned medium–induced gemcitabine uptake in MCA/129 tumor cells in the second phase of the assay (Supplemental Figure 5E).

We previously showed that a short-acting antiangiogenic drug administered 1 hour before single-dose radiotherapy or specific chemotherapies to transiently de-repress ASMase signaling leads to enhanced ceramide-mediated vascular injury and improved xenograft response (8). Using the same principle, MCA/129 tumor-bearing athymic nude mice were pretreated for 1 hour with the well-characterized anti-VEGFR2 mAb DC101 (1.6 mg/25 g mice, i.v.) to de-repress ASMase; [^3^H]gemcitabine (240 mg/kg “cold” gemcitabine plus [^3^H]gemcitabine as “tracer”) was administered i.p.; and 1 hour thereafter when tumor steady-state gemcitabine uptake was reached, tumors were collected and gemcitabine uptake was quantified by liquid scintillation counting. We observed that DC101 pretreatment increased gemcitabine uptake into MCA/129 tumors by 3-fold, accompanied by quantitative reduction in serum gemcitabine level (Figure 5, D and E), an event blocked by the ENT1 inhibitor dilazep (20 mg/kg, i.p.) (Supplemental Figure 6). Consistent with these data, timed delivery of DC101 at 1 hour prior to, but not concomitant with, gemcitabine enhanced MCA/129 fibrosarcoma endothelial cell apoptosis (Figure 6A) (*P* < 0.05) and tumor response (Figure 6B) (*P* < 0.001).

To confirm antiangiogenic chemosensitization is mediated by ENT1 functionalization in ASMase-induced CRPs, mice harboring MCA/129 fibrosarcomas were pretreated before each gemcitabine dose with the ASMase inhibitor imipramine (10 mg/kg injected i.p. twice on the day before and on the day of gemcitabine treatment) or anti-ceramide 6B5 scFv (150 μg/mouse s.c. at 10 minutes prior to DC101), both of which inhibit DC101 chemosensitization (Figure 6, C and D, respectively). Similarly, siRNA knockdown of ENT1 in MCA/129 fibrosarcoma cells (Figure 6E), which inhibits bSMase enhancement of gemcitabine-induced apoptosis in vitro (Figure 6F), abrogated DC101 chemosensitization of the gemcitabine effect in vivo (Figure 6G), reverting the overall gemcitabine response to the modest impact observed at baseline.

These studies suggest that advanced sarcoma, a cancer with poor outcomes, might be unresponsive to gemcitabine treatment because the drug fails to engage CRP signaling and effectively cross tumor-cell plasma membranes. This potentially is treatable by strictly timed delivery of an antiangiogenic agent to facilitate gemcitabine-induced endothelial cell ASMase secretion.

### Substantial volumetric changes were observed in a prospective clinical trial in sarcoma that included a 1-hour pretreatment period of bevacizumab.

To explore engagement of endothelial ASMase in clinical tumor chemotherapy, an ongoing prospective phase II clinical trial of gemcitabine and docetaxel with the antiangiogenic drug bevacizumab in advanced soft tissue sarcoma, conducted at Memorial Sloan Kettering Cancer Center (MSKCC) (MSKCC IRB 09-015; ClinicalTrials.gov identifier NCT00887809), was addended to incorporate a 1-hour pretreatment period of bevacizumab before the administration of chemotherapy in a cohort of participants (Figure 7A). Patients eligible for the trial had metastatic or recurrent leiomyosarcoma, undifferentiated pleomorphic sarcoma, pleomorphic liposarcoma, pleomorphic rhabdomyosarcoma, or angiosarcoma (Figure 7B). The study was initially a randomized, double-blind study of gemcitabine and docetaxel, combined with either bevacizumab or matching placebo. Because of poor accrual, the study was modified into a single-arm, open-label study in which all participants received gemcitabine, docetaxel, and bevacizumab. Participants were treated on day 1 of each 21-day cycle with bevacizumab (15 mg/kg) delivered i.v. over 30 minutes, followed by i.v. administered gemcitabine (900 mg/m^2^ over 90 minutes). On day 8, gemcitabine (900 mg/m^2^ over 90 minutes) was administered, followed by docetaxel (75 mg/m^2^ over 60 minutes), without bevacizumab.

When the preclinical animal data we have reported thus far became available, the protocol underwent an additional amendment that changed timing of the day 1 bevacizumab from immediately before gemcitabine to 1 hour before gemcitabine administration to allow time for improved transport of the drug. To facilitate comparison with our preclinical data, a post hoc volumetric analysis of tumor response was performed after the trial had completed accrual. This was conducted by a musculoskeletal radiologist blinded to assignment of patients to the bevacizumab-gemcitabine administration schedule, as detailed in Methods. Baseline CT imaging or MRI was used to identify a dominant target lesion for volumetric measurement, followed by volumetric assessment on initial post-treatment follow-up scan (at 2 months after initiating treatment) and over the treatment course (a representative CT time course is shown in Figure 7C). A total of 44 patients were treated during the trial. Three patients who received a combination of both treatment schedules and another 3 patients who did not have evaluable imaging studies were excluded from the analysis. Of the 38 remaining patients, 16 were treated on the immediate bevacizumab-gemcitabine schedule, 14 were on the 1-hour interval schedule, and 8 received placebo rather than bevacizumab. Although the study was not designed to compare the various treatment groups, and subsequent statistical analyses were not included in the original trial design, the volumetric data are hypothesis-generating. Six of 16 patients (38%) who received immediate consecutive bevacizumab-gemcitabine delivery had a substantial volumetric response (defined as ≥30% reduction in tumor volume), whereas nearly all patients (*n* = 13 of 14; 93%) in the bevacizumab-gemcitabine 1-hour-interval-schedule cohort had a marked volumetric response (Figure 7D) (*P* = 0.0017 by 2-tailed Fisher’s exact test and *P* = 0.005 by the Mann-Whitney rank-sum test). Differences between consecutive bevacizumab-gemcitabine and 1-hour delayed delivery also occurred at other levels of volume reduction, with 50% volume reduction occurring in 38% of the immediate and 79% of the 1-hour-interval-schedule group (*P* = 0.03) and 80% volume reduction in 13% of the immediate and 50% of the 1-hour-interval-schedule group (*P* = 0.04).

As reported elsewhere, the study had a high early censor rate because many patients in the immediate timing discontinued participation in the study early for reasons other than disease progression. This makes longitudinal comparisons between the 2 groups difficult (49). Therefore, volumetric analysis was also performed on the initial post-treatment follow-up scan to evaluate an early time point available for all patients (Supplemental Figure 7). On the initial treatment follow-up scan, 4 of 16 patients (25%) who received immediate consecutive bevacizumab-gemcitabine delivery had a reduction of tumor volume of ≥30%, whereas 9 of 14 (64%) in the bevacizumab-gemcitabine 1-hour-interval-schedule cohort had ≥30% volumetric reduction (*P* = 0.0075 by Mann-Whitney rank-sum test; *P* = 0.0362 by 1-sided 2-sample proportion test). A trend toward significance was also observed at 50% tumor volume reduction (*P* = 0.0773 by 1-sided 2-sample proportion test).

## Discussion

This study redefines the mechanism of ENT1-mediated nucleoside drug transport, which has long been considered constitutive. We demonstrate gemcitabine uptake into sarcoma cells is a 2-step process involving ASMase-induced ceramide-mediated CRP formation on the cell surface and the insertion of ENT1 for functionalization. Whether ENT1 exists in a loosely bound dimerized state prior to insertion, which is then strengthened upon insertion, or whether it dimerizes within the CRP is currently unknown. Based on studies by Pin Lan Li et al. (50), however, which show that the 6 subunits of NADPH oxidase 2, 4 of which are cytosolic and 2 of which are membrane bound, oligomerize and functionalize superoxide generation within a CRP, we favor the latter concept. In either case, secretion of ASMase by tumor cells, if available, or as a bystander event by neo-angiogenic microvasculature would be requisite for such oligomerization. It is noteworthy that the preclinical data showing precisely timed delivery of bevacizumab to reverse VEGF inhibition of bystander ASMase-mediated ENT1 drug transport in sarcoma xenografts are recapitulated in human sarcomas in a prospective phase II clinical trial. This not only validates the relevance of the preclinical model dataset but strongly supports ENT1 dimerization or oligomerization as a principal determinant of clinical drug response.

Targeting upstream elements of our proposed molecular ordering of ENT1 dimerization, pharmacologically using imipramine to inhibit the ASMase bystander effect or anti-ceramide scFv to prevent CRP formation, or genetically using ENT1 siRNA, abrogates DC101 chemosensitization without affecting the modest baseline gemcitabine effect observed in MCA/129 xenografts. We interpret these data as consistent with gemcitabine uptake in tumors occurring by 2 distinct mechanisms: an ENT1-independent baseline mechanism, which is marginally effective, perhaps via concentrative nucleoside transporters; and an ENT1-dependent mechanism that is highly effective although suppressed by VEGF in the tumor microenvironment and reversible by timed delivery of an antiangiogenic drug. Additional investigation will be required to elucidate this biological program.

Effectively engaging the CRP biology described here presents several opportunities for improvement in therapeutic index. First, we have evidence that antiangiogenic sensitization of tumor response is specific to neo-angiogenic microvasculature, which is under VEGF regulation in the hypoxic tumor microenvironment. In this regard, strictly timed delivery of DC101 also enhances MCA/129 fibrosarcoma endothelial cell apoptosis and tumor response to high single-dose radiotherapy (51) without affecting radiation-induced endothelial cell apoptosis in the normoxic C57BL/6J mouse small intestines or consequent lethality from the gastrointestinal acute radiation syndrome (52), 1 of 2 lethal radiotoxicities that might occur after a nuclear catastrophe (40, 53). Assuming other normoxic normal tissues display the same phenotype, it might be possible, by selectively enhancing ASMase/ceramide-mediated tumor cell membrane reorganization, to promote gemcitabine uptake in multiple primary and metastatic settings without added toxicity. Supplemental Table 1 provides a comprehensive list of human cancers in which gemcitabine is currently indicated for clinical use. This list defines an extensive and diverse set of tumor types for which gemcitabine is delivered either as single agent or in combination therapy. Hence, if ASMase-CRP-ENT1–mediated gemcitabine transport can be enhanced generically, it might have broad application for cancer therapy. An extension of this concept is that it might be possible to expand gemcitabine use to cancers for which it is not currently effective if sufficient increase in drug uptake were to be achieved by engaging bystander ASMase secretion.

Our recent studies point to another avenue for potential improvement of the gemcitabine therapeutic index based on concepts explored here. We identify a theoretical problem that antiangiogenic drugs such as bevacizumab, an IgG with a 3-week half-life, might accumulate progressively during the multiple 3-week chemotherapeutic cycles that currently are standard of care for soft tissue sarcoma treatment (8). In this context, we published evidence that antiangiogenic ASMase-dependent enhancement of the response of MCA/129 fibrosarcomas implanted in sv129/BL6 mice to gemcitabine progressively diminishes as the level of the VEGFR2 inhibitor DC101, an IgG, accumulates, suggesting a short-acting antiangiogenic drug might be preferable in multicycle chemotherapeutic regimens (8). Consistent with this concept, comparison of volumetric responses of soft tissue sarcomas in the 1-hour delayed bevacizumab treatment cohort (Figure 7D) with the response at the time of the first scan after 2 cycles of chemotherapy (Supplemental Figure 7) suggests there might be a waning of tumor response over time as drug accumulates. Last, an additional potential benefit of a short-acting antiangiogenic drug in sarcoma treatment might be the capability of delivering it twice during each 3-week chemotherapy cycle to enhance the action of gemcitabine, which is administered on days 1 and 8 of the regimen used in the phase 2 trial reported here. In this regard, we are currently in the late stages of development of such a short-acting antiangiogenic agent to test these concepts clinically.

Whether other nucleoside chemotherapeutics use this ASMase/ENT1 mechanism to maximize uptake is currently unknown, although it is an obvious question. There are many nucleoside drugs approved for clinical use (54, 55). Nucleoside drugs fall into 2 broad categories: anticancer agents and antiviral agents. Supplemental Table 2 provides a list of 10 clinically approved drugs in each category. Although we have no data addressing whether antiviral nucleosides dimerize in CRPs for optimal transmembrane transport, we have generated preliminary data that show the nucleoside chemotherapeutic capecitabine, like gemcitabine, induces marked ENT1-ENT1 homodimerization within seconds in the HEK293 assay (AG. and RK, unpublished observation). This latter finding implies that ENT1 homodimerization in CRPs may represent a general mechanism for optimal nucleoside chemotherapeutic drug uptake. An additional question of broad interest is whether physiologic nucleosides use this feed-forward, sphingolipid-based mode of transport, and, if so, under what conditions is this system engaged. We are currently in the process of addressing these unanswered questions within our program.

Using principles learned in preclinical models regarding ASMase/ENT1 signal transduction in a phase 2 clinical trial for advanced sarcoma resulted in a higher-than-anticipated volumetric response rate. Although the impact of precise temporal antiangiogenic delivery on gemcitabine treatment of advanced sarcoma appears encouraging in our clinical analysis, the extent to which precise temporal antiangiogenic timing might chemosensitize human cancer warrants further investigation. We propose that if temporal restriction for sphingolipid-based antiangiogenic chemosensitization can be validated in a larger randomized clinical trial, this paradigm would represent a substantive advance in the way we use antiangiogenic drugs in cancer therapy.

## Methods

### Sex as a biological variable.

Male mice were used for all experiments. Sex was not considered a biological variable, because findings were expected to generalize across sexes based on prior mechanistic data obtained in similar tumor response models.

### Cell culture.

BAECs were established from the intima of bovine aorta, as described previously (56, 57). Cells were grown in 100-mm dishes in DMEM (Hyclone) low-glucose medium with 10% bovine calf serum, penicillin (50 units/mL), and streptomycin (50 μg/mL). At 3 days after confluence, cells exhibited features of contact-inhibited monolayers. These growth-arrested cells were either used for experiments or underwent additional sub-culturing (up to passage 18), split at a ratio of 1:6. For sub-culturing, monolayers were dissociated by trypsinization (TrypLE, Hyclone) for 2–3 minutes at 37°C, washed twice, and resuspended in DMEM with the same supplements listed earlier in this paragraph. BAECs were maintained at 37°C in 10% CO_2_ humidified incubators. MCA/129 fibrosarcoma cells were maintained under log growth conditions in DMEM high-glucose medium supplemented with 10% FBS, penicillin (100 units/mL), and streptomycin (100 μg/mL) at 37°C.

### Materials and reagent.

Gemcitabine (Mylan) was procured from the MSKCC pharmacy and stored at room temperature per the manufacturer’s instruction. Mouse IgM monoclonal anti-ceramide Ab MID 15B4 (catalog ALX-804-196) was from Enzo Life Science. Rabbit polyclonal Ab to ENT1 was from Abcam (catalog ab135756). Mouse monoclonal anti-GAPDH (Santa Cruz Biotechnologies, sc-47724; dilution 1:1,000) and mouse monoclonal anti–α-tubulin (Abcam, ab7291; dilution 1:10,000) Abs were used for Western blots. Alexa Fluor 488–conjugated goat anti-rabbit (catalog 111-545-003) and Cy3-conjugated goat anti-mouse secondary Abs (catalog 115-165-003) were procured from Jackson ImmunoResearch. Bacterial SMase (*Bacillus cereus*, S9396) was purchased as a 371 units/mL stock in 50% glycerol from Sigma-Aldrich. The ENT1 inhibitors NBMPR (catalog N2255) and dilazep (catalog D5294) were used at 100 nM and 10 μM for cell culture and 20 mg/kg i.p. for mouse studies, respectively, and procured from Sigma-Aldrich. Recombinant human VEGF-165 (R&D Systems, 293-VE) was used at 2 ng/mL. Imipramine hydrochloride (catalog I7379) and methyl β-cyclodextrin (catalog M7439) were procured from Sigma-Aldrich and used at 50 μM and 5 mM concentrations, respectively. Anti-ceramide 6B5 scFv was purchased from LakePharma.

### Gemcitabine-uptake studies.

Gemcitabine-uptake studies were performed in 24-well plates with cells grown to ~50% to 60% confluence (proliferative phase or log growth phase) or at 3 days after 100% confluence (growth-arrested phase) for BAECs, and at confluence for MCA/129 cells. Cells maintained overnight in 0.2% HSA containing serum-free medium, were exposed to 10 μM gemcitabine with ^14^C-gemcitabine as tracer (Moravek Inc.; specific activity 55 mCi/mmol; 0.1 mCi/mL in a total volume of 1 mL) for different time points (5 seconds to 5 minutes). Wells were rinsed with PBS and cells were lysed in lysis buffer (1% SDS, 0.3% sodium hydroxide). Cell-associated radiolabel was quantified by a TriCarb 2910 TR liquid scintillation counter (PerkinElmer) using ScintiVerse BD Cocktail (Thermo Fisher Scientific). Triplicate parallel wells were used to quantify cell numbers by hemocytometer after trypsinization.

### Confocal microscopy.

BAECs and MCA/129 cells were grown on 6-well plates. Cells were incubated overnight in 0.2% HSA containing serum-free medium and then treated with gemcitabine with or without conditioned medium obtained from BAECs treated with or without gemcitabine for 1 minute. Cells were then fixed for 10 minutes in fresh 2% paraformaldehyde, washed twice with cold PBS, and blocked with 2% normal donkey serum (D9663, Sigma) in PBS for 1 hour on ice. Cells were stained with anti-ceramide MID 15B4 IgM (Alexis Corp.) at a 1:50 dilution and/or anti-rabbit ENT1 (Abcam) overnight at 4°C, washed 3 times in PBS and thereafter with anti-mouse Cy3 and/or anti-rabbit Alexa Fluor 488–conjugated secondary Ab (Jackson ImmunoResearch Laboratories), respectively, at a 1:500 dilution for 1 hour at 4°C. Cells were washed 3 times in PBS and mounted in Vectashield antifade mounting medium containing DAPI (H-1000-10, Vector Laboratories). CRPs were imaged on a Leica SP5 Inverted microscope with a HyD hybrid detector photon counter and HCX PL APO CS 63.0×/1.40 oil objective lens with digital zoom 7.

### 3D volumetric analysis.

Volumetric analysis was performed using Imaris software (Bitplane). Briefly, 10 individual *Z*-stacks for each cell were acquired under system optimized conditions (*Z*-step size of 0.13 μm), and data were analyzed using Imaris software (Bitplane). Intensity normalization was performed for control and treated samples. Briefly, the intensity mean threshold was obtained from control samples. Using the intensity mean threshold, surfaces were created for control and treated cells using the “Surfaces” feature in Imaris software. Intensity mean threshold allows staining intensity values above a mean threshold to be considered as positive for surface volume quantification, whereas values below this range are considered negative. Based on the positive intensity mean value for each cell, surface volumes are generated. Individual volumes for each cell are then summed and mean surface volume is calculated for each sample set. Animated visualization of 3D data sets can be exported as movies, using the key frame animation function in Imaris.

### ENT1 siRNA transfection.

MCA/129 cells were transfected with siRNA targeting ENT1 (500 nM; 10620319, Invitrogen) using Lipofectamine 3000 reagent (Thermo Fisher Scientific), following a modified protocol based on previously published methods (58, 59) and the manufacturer’s recommendation. As a negative control, cells were transfected with a nontargeting siRNA containing a scrambled nucleotide sequence. Transfection was performed in serum-free medium, and cells were used for downstream experiments 48 hours after transfection. Efficiency of ENT1 knockdown was monitored by Western blotting to quantify protein-level suppression.

### Apoptosis in vitro.

Cells were harvested at 16 hours after treatment. Detached cells and cells suspended in medium were pooled, and total cells were collected by centrifugation at 270*g* for 5 minutes. After washing twice with PBS, cells were fixed with 2% paraformaldehyde, stained using bis-benzimide trihydrochloride (Hoechst 33258; Sigma-Aldrich) as described elsewhere (60), and placed on glass slides for counting on an Olympus IMT-2 microscope using a ×40 DPlanApo40UV 0.850160/0.11-0.23 objective lens at room temperature. Apoptosis was quantified based on morphologic characteristics of apoptotic nuclei, including chromatin condensation, segmentation, and compaction, along with nuclear periphery and the appearance of apoptotic bodies. A minimum of 100 cells were counted per sample.

### ASMase activity assay.

ASMase activity was quantified by radioenzymatic assay using bovine [^14^C-methylcholine]sphingomyelin (PerkinElmer) as the substrate, as described previously (7, 61). Cells were incubated in serum-free medium for 4 hours prior to study. For cellular ASMase activity, cells were washed with PBS and then lysed with 0.5% Triton X-100. The amount of cellular protein was determined by DC protein assay (Bio-Rad). For secreted ASMase activity, the cell media were pooled, and protein was concentrated using Amicon Ultra-4 centrifugal filters (Merck Millipore) by a single centrifugation step at 3,200*g* for 15 minutes. Briefly, a film of unlabeled bovine brain sphingomyelin (Avanti Polar Lipids) and [^14^C]sphingomyelin (39:1 molar ratio) was hydrated in a 3× acidic assay buffer (1% Triton X-100, 250 mM sodium acetate, pH 5.0, 128 mM SM). Zinc chloride (0.1 mM) was added to measure total ASMase, and 5 mM EDTA was added to measure zinc-independent ASMase. Radiolabeled buffer (30 μL) was added to 60 μL of sample (either concentrated medium or 25 mg of cell lysate) to initiate the reaction. After 2 hours of incubation at 37°C, the reaction was terminated by adding 125 �L of a 2:1 (vol/vol) chloroform-to-methanol solution. Samples were well mixed and centrifuged for 5 minutes at 7,000*g*. The aqueous (upper) phase was transferred into scintillation vials containing ScintiVerse BD Cocktail (Thermo Fisher Scientific) for quantitation using a 2200CA Tri-Carb scintillation counter (Packard Instrument). For each assay, a buffer only and a sample boiled for 5 minutes at 95°C were included as negative controls.

### NanoBiT protein-protein interaction studies.

The NanoBiT system (Promega) is designed to detect protein-protein interactions registered as luminescence. The principle of the system is that 2 subunits — large BiT and small BiT — are fused to proteins of interest, which form an enzyme upon protein-protein interaction that generates light detected using a luminometer. Here, we used this system to quantify ENT1-ENT1 interaction (dimerization) after gemcitabine treatment in live cells, as published by Grañe-Boladeras et al. (48). ENT1, subcloned into the N-terminus of large and small BiT vectors, were provided by Imogen Coe (Toronto Metropolitan University, Toronto, Ontario, Canada). The manufacturer protocol is at https://www.promega.com/products/protein-interactions/live-cell-protein-interactions/nanobit-ppi-starter-systems/?catNum=N2014#protocols

HEK293 cells (*n* = 5,000 cells/well) were plated in a 96-well plate; at 70% to 80% confluence, cells were transfected with plasmids (100 ng of large-BiT ENT1 and small-BiT ENT1 each) using Lipofectamine 3000 reagent (Thermo Fisher Scientific), according to manufacturer’s instructions. After 48 hours, the Nano-Glo live assay system (Promega) was used to quantify protein-protein interactions, per the manufacturer’s protocol. Briefly, complete medium in 96-well plates was aspirated and replaced with 100 �L of Opti-MEM reduced serum medium. Gemcitabine (10 μM) was added to each well, the reaction was stopped by placing cells on ice, and Nano-Glo live cell reagent was added to each well and gently mixed by hand. Immediately, luminescence was measured on a Glomax 96 Microplate Luminometer (Promega, 9101-002).

### Bystander effect studies.

BAECs were treated with 10 μM gemcitabine for 10 minutes. Control and treated media were pooled and centrifuged at 3,200*g* for 15 minutes and concentrated in a 30,000 molecular weight cutoff Amicon Ultra-4 centrifugal unit (Merck Millipore) to remove gemcitabine but retain secreted ASMase. In some studies, exogenous gemcitabine was added back to the pooled medium before treating MCA/129 cells.

### Gemcitabine uptake in vivo.

For gemcitabine-uptake studies, male athymic nude mice (Envigo; Hsd Athymic nude-Foxn1^nu^ strain) were implanted with MCA/129 tumors (2 × 10^6^ cells/25 g mouse). Mice harboring tumors at 100–150 mm^3^ were treated with DC101 (1.6 mg/25 g mouse i.v.) 1 hour prior to treatment with gemcitabine (240 mg/kg “cold” gemcitabine plus ^3^H-gemcitabine tracer, i.p.). Animals were euthanized after 1 hour, tumors were excised, and blood was collected. Tumors were then solubilized using Solvable Tissue Solubilizer (PerkinElmer, 6NE9100) and heating in a water bath for 2 hours at 60°C. Dissolved tissue was allowed to cool at room temperature for 30 minutes before adding 0.2–0.5 mL hydrogen peroxide to complete solubilization. Gemcitabine uptake into solubilized tumor tissue was calculated by scintillation counting, as described under *Gemcitabine-uptake studies*. Serum gemcitabine content was quantified after allowing blood to clot for 15–20 minutes, centrifugation at 2,000*g* for 10 minutes at 4°C, and scintillation counting. For inhibitor studies, dilazep hydrochloride (20 mg/kg, i.p.), dissolved in 0.9% saline solution, was injected with DC101 at 1 hour before gemcitabine treatment.

### Tumor models.

MCA/129 murine sarcoma cells were maintained in DMEM with 10% FBS containing 100 units/mL penicillin and 100 μg/mL streptomycin. Cells were grown as monolayers in 75 cm^2^ culture flasks at 37°C in 10% CO_2_ humidified incubators. Cells in log growth were trypsinized, washed in PBS, and diluted in Matrigel/PBS solution (40:60 vol/vol) for MCA/129 syngeneic tumor grafts. Cells (1 × 10^6^) were injected s.c. into the flanks of mice, as described previously (62).

### Tumor response in vivo.

sv129/BL6 mice harboring MCA/129 tumors (100–150 mm^3^) were treated with a maximally tolerated regimen of 3 i.p. injections of 240 mg/kg gemcitabine on a biweekly schedule with or without i.v. DC101. DC101 (BE0060, Bio X Cell), a rat monoclonal anti-mouse VEGFR2 IgG, was administered at 1,600 μg/25 g mouse via tail vein injection 1 hour prior to gemcitabine administration (8, 52). Anti-ceramide 6B5 scFv, generated as previously described (40, 63), was purchased from LakePharma. Tumor volume, based on caliper measurements, was calculated daily according to the formula of Kim et al. (64).

### Inhibition of tumor response with imipramine, anti-ceramide 6B5 scFv, and ENT1 siRNA.

Male athymic nude mice (Envigo; Hsd Athymic nude-Foxn1nu strain) were implanted with MCA/129 tumor cells in the right flank (1 × 10^6^ cells/mouse). Mice harboring 75–100 mm^3^ tumors received i.p. injections of imipramine hydrochloride (Sigma-Aldrich) dissolved in water (10 mg/kg) twice daily, starting 1 day before initiation of gemcitabine therapy and continuing through the day of gemcitabine administration for a total of 3 DC101 plus gemcitabine treatment doses. For anti-ceramide scFv studies, athymic nude mice bearing established tumors were administered anti-ceramide 6B5 scFv at a dose of 150 μg/mouse via s.c. injection. Treatment was initiated 10 minutes prior to administration of DC101 and continued for a total of 3 doses. For ENT1 siRNA studies, MCA/129 cells were transfected with either ENT1 siRNA or nontargeting control siRNA using Lipofectamine 3000 reagent (Thermo Fisher Scientific), following the manufacturer’s protocol for 48 hours. Thereafter, efficiency of ENT1 knockdown was monitored by Western blotting to quantify protein-level suppression before implanting into animals.

### Apoptosis in vivo.

To evaluate acute endothelial apoptosis, tumors were grown in mice and treated with drugs, as we have described. After the first treatment, tumors were harvested at specified times and processed according to the report by Garcia-Barros et al. (62). Paraffin-embedded 5 μm sections were co-stained for endothelial apoptosis using TUNEL and the endothelial specific mAb MECA-32 (Developmental Studies Hybridoma Bank, developed under the auspices of the *Eunice Kennedy Shriver* National Institute of Child Health and Human Development and maintained by The University of Iowa; MECA-32-s, dilution 1:10).

### Phase II trial of gemcitabine and docetaxel with bevacizumab in selected sarcoma subtypes.

Patients with metastatic or locally recurrent leiomyosarcoma, pleomorphic undifferentiated sarcoma, pleomorphic liposarcoma, rhabdomyosarcoma, or angiosarcoma were treated during a phase II trial of gemcitabine, docetaxel, and bevacizumab. Initially the trial was a double-blind, placebo-controlled, randomized trial of gemcitabine and docetaxel with or without bevacizumab, which was modified to a single-arm, open-label, nonrandomized study of gemcitabine, docetaxel, and bevacizumab (ClinicalTrials.gov identifier NCT00887809). On day 1 of each 21-day cycle, patients were treated with bevacizumab 15 mg/kg i.v. over 30 minutes, with gemcitabine 900 mg/m^2^ over 90 minutes on days 1 and 8, and docetaxel 75 mg/m^2^ over 60 minutes on day 8. Initially, gemcitabine was administered immediately following bevacizumab. However, based on the results of our preclinical studies, the protocol was later amended so that gemcitabine was administered at 1 hour after bevacizumab.

Volumetric measurement of tumors was performed on an exploratory basis because the original study was not designed or powered to specifically investigate the clinical application of timed antiangiogenic therapy as a method of tumor chemosensitization. Considering the results from our animal studies, which became available partway through the clinical study period, volumetric measurements were proposed to facilitate comparison with our preclinical data. Furthermore, they likely provide a more meaningful assessment of tumor response than the Response Evaluation Criteria in Solid Tumors–based evaluation for metastatic soft tissue sarcoma (65). Therefore, on completion of the study, volumetric analysis was performed by a study radiologist blinded to assignment of patients to the bevacizumab administration schedule or the initial placebo cohort. Briefly, baseline CT imaging or MRI was used to identify a dominant target lesion for volumetric measurement. The most representative lesion was chosen. This was generally the largest lesion with well-defined borders. The outer border of the representative lesion was traced on every CT slice. The area was then calculated for each slice. The sum of the slice areas for each lesion multiplied by the slice thickness (generally 5 mm) to create a volumetric measurement for the chosen tumor. This process was then repeated for the nadir lesion, which was determined by evaluating each scan during the study period. Tumor response was defined at the levels of 30%, 50%, and 80% reduction in tumor volume. Both a 2-tailed Fisher’s exact test and the Mann-Whitney rank-sum test were used to evaluate significance in patient volumetric response rates at the first interval treatment scan (week 6) and at time of maximum treatment response.

### Statistics.

The data are presented as mean ± SEM. Differences between the 2 groups were analyzed using an unpaired 2-tailed *t* test. For comparisons involving more than 2 groups, 1-way ANOVA with Tukey’s post hoc test for multiple comparisons was performed. Statistical analyses were performed with Prism 10 software (GraphPad Software). Waterfall plots for clinical trial data were analyzed by 2-tailed Fisher’s exact test and the Mann-Whitney rank-sum test. *P* < 0.05 was considered statistically significant.

### Study approval.

Mice were housed at the Research Animal Resource Center of MSKCC, a facility approved by the American Association for Accreditation of Laboratory Animal Care and maintained in accordance with the regulations and standards of the US Department of Agriculture and the NIH of the US Department of Health and Human Services. All animal experimental protocols were approved by the MSKCC Research Animal Resource Center and the IACUC. For the nonrandomized phase II trial of gemcitabine, docetaxel, and bevacizumab, the protocol was approved by the MSKCC IRB, and all patients provided written informed consent (ClinicalTrials.gov identifier NCT00887809).

### Data availability.

All data are available in the main text, the supplement, or the Supporting Data Values file. Plasmids are available upon establishment of a material transfer agreement with MSKCC. All other data supporting the findings of this study are available from the corresponding author upon reasonable request.

## Author contributions

AG, GS, ZF, and RK contributed to the study design. AG, SR, RAL, CT, JC, GS, and RK contributed to conducting experiments, acquiring data, and analyzing data. AG, JC, and KM conducted the basic investigation. SR, MAD, RAL, CT, GS, WDT, and ZZ conducted the clinical trial. AHF, GS, ZF, and RK provided reagents. AG, AHF, GS, ZF, WDT, and RK wrote the original draft of the manuscript and reviewed and edited the manuscript.

## Conflict of interest

RK reports patents (US7195775B1, US7850984B2, US10052387B2, US10414533B2, US20140205543A1), as do RK and JAR (US8562993B2, US9592238B2, US10450385B2, US20150216971A1, US20170335014A1, US20190389970A1, and US20190046538A1); RK and ZF report patents (US20170333413A1 and US20180015183A1); and ZF reports a patent (US10413533B2). RK and ZF are cofounders of Ceramedix Holding.

## Funding support

This work is the result of NIH funding, in whole or in part, and is subject to the NIH Public Access Policy. Through acceptance of this federal funding, the NIH has been given a right to make the work publicly available in PubMed Central.

- Mr. William H. Goodwin and Mrs. Alice Goodwin and the Commonwealth Foundation for Cancer Research and The Center for Experimental Therapeutics (grant GC238047) at Memorial Sloan Kettering Cancer Center.
- NIH National Cancer Institute (NCI) (grants R21 CA198586 and R01 CA255336 to RK).
- NIH NCI Cancer Center Support Core (grant P30 CA008748 to MSKCC).

## Supplementary Material

Supplemental data

Unedited blot and gel images

Supplemental video 1

Supplemental video 2

Supporting data values

## Figures and Tables

**Figure 1:**
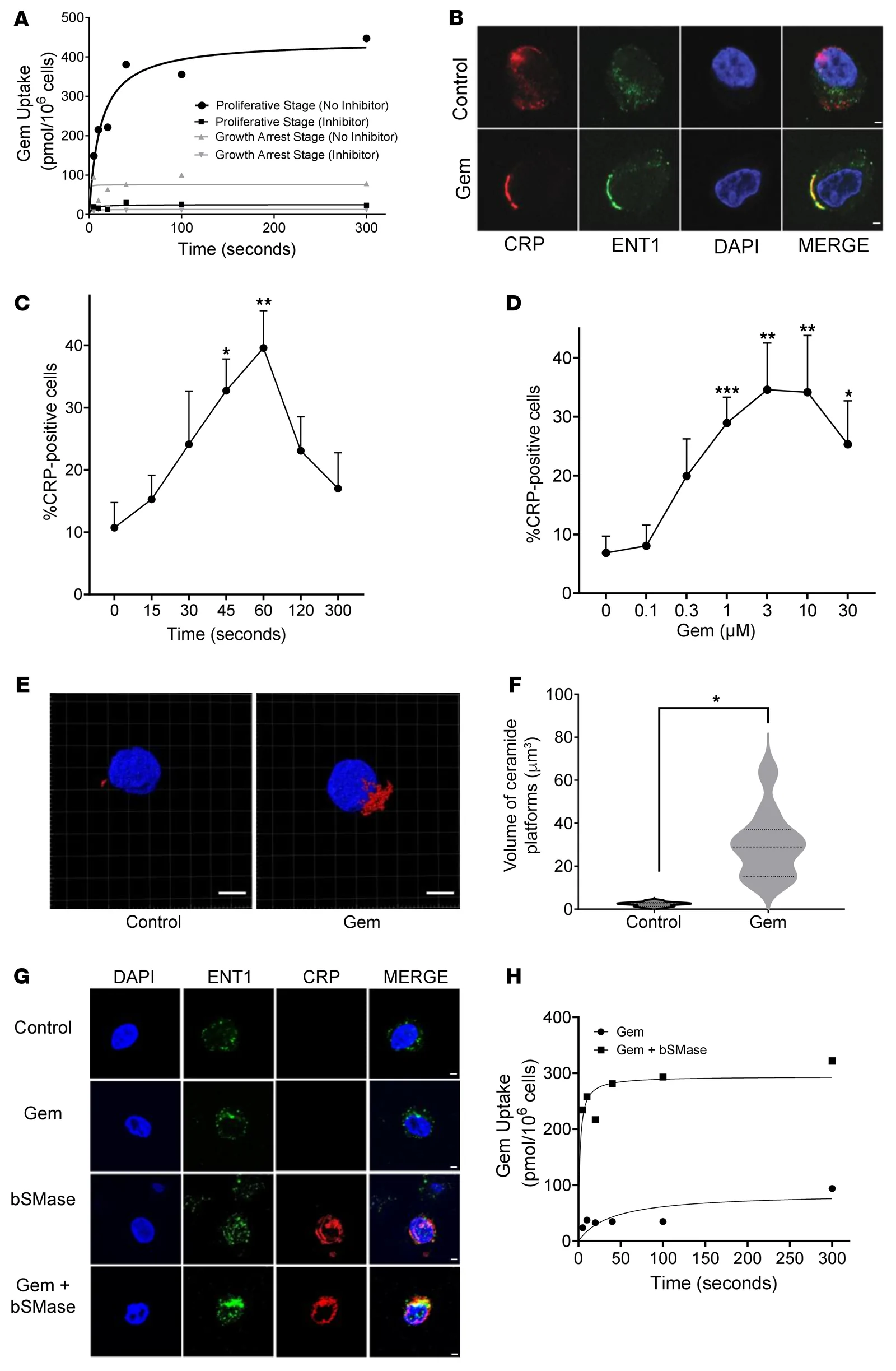
Nucleoside transporter ENT1 requires CRPs for functionalization of gemcitabine uptake. (**A**) Uptake of gemcitabine (10 μM gemcitabine plus tracer [^14^C] gemcitabine) in proliferative and growth-arrested BAECs was performed in the presence and absence of ENT1 inhibitors (100 nM NBMPR and 10 μM dilazep). Proliferative cells were 50%–60% confluent; growth-arrested cells were used 3 days post-confluence. Inhibitors were delivered 1 hour before gemcitabine. Cell-associated radioactivity was quantified by scintillation counting. Shown is 1 representative of 2 independent experiments performed in triplicate. (**B**) Confocal images showing colocalization of ENT1 in a CRP 1 minute after 1 µM gemcitabine. Nuclei were stained with DAPI (blue), ENT1 with rabbit anti-ENT1 followed by Alexa Fluor 488–conjugated anti-rabbit IgG (green), and ceramide with mouse anti-ceramide MID 15B4 followed by Cy3-conjugated anti-mouse IgM (red). Merged images indicate ENT1 localization in CRPs. Data are representative of 10 cells/group from 1 of 2 independent experiments. (**C**) Time course of gemcitabine-induced CRP formation (1 μM) in BAECs. Data represent the mean ± SEM. *n* = 2. **P < 0.01, *P < 0.05 vs. 0-second point by 1-way ANOVA. (**D**) Dose response of gemcitabine-induced CRP formation in BAECs at 1 minute (gemcitabine dose 0.1–30 μM). Data represent the mean ± SEM. *n* = 2. ***P < 0.001, **P < 0.01, *P < 0.05 vs. 0 μM by 1-way ANOVA. (**E**) 3D still image of CRPs in control and gemcitabine-treated cells at 1 minute after 1 μM gemcitabine treatment in proliferative BAECs. The 3D still image was acquired using Imaris software by generating *Z*-stacks during the image acquisition as described in Methods. (**F**) CRP volumes from **E**. Data represent mean ± SEM from 2 independent experiments using 10 cells/sample. Two-tailed Student’s *t* test. *P < 0.05. (**G**) Confocal images showing CRP formation and ENT1 localization in growth-arrested BAECs treated with gemcitabine and/or bSMase (*Bacillus cereus*; 5 units/well). Cells were stained as in **B**. Data are representative of 10 cells/group from 1 of 2 independent experiments. (**H**) Gemcitabine uptake in growth-arrested BAECs pretreated with bSMase (5 units/well). Cells were processed as in **A**. Data are representative of 3 independent experiments.

**Figure 2:**
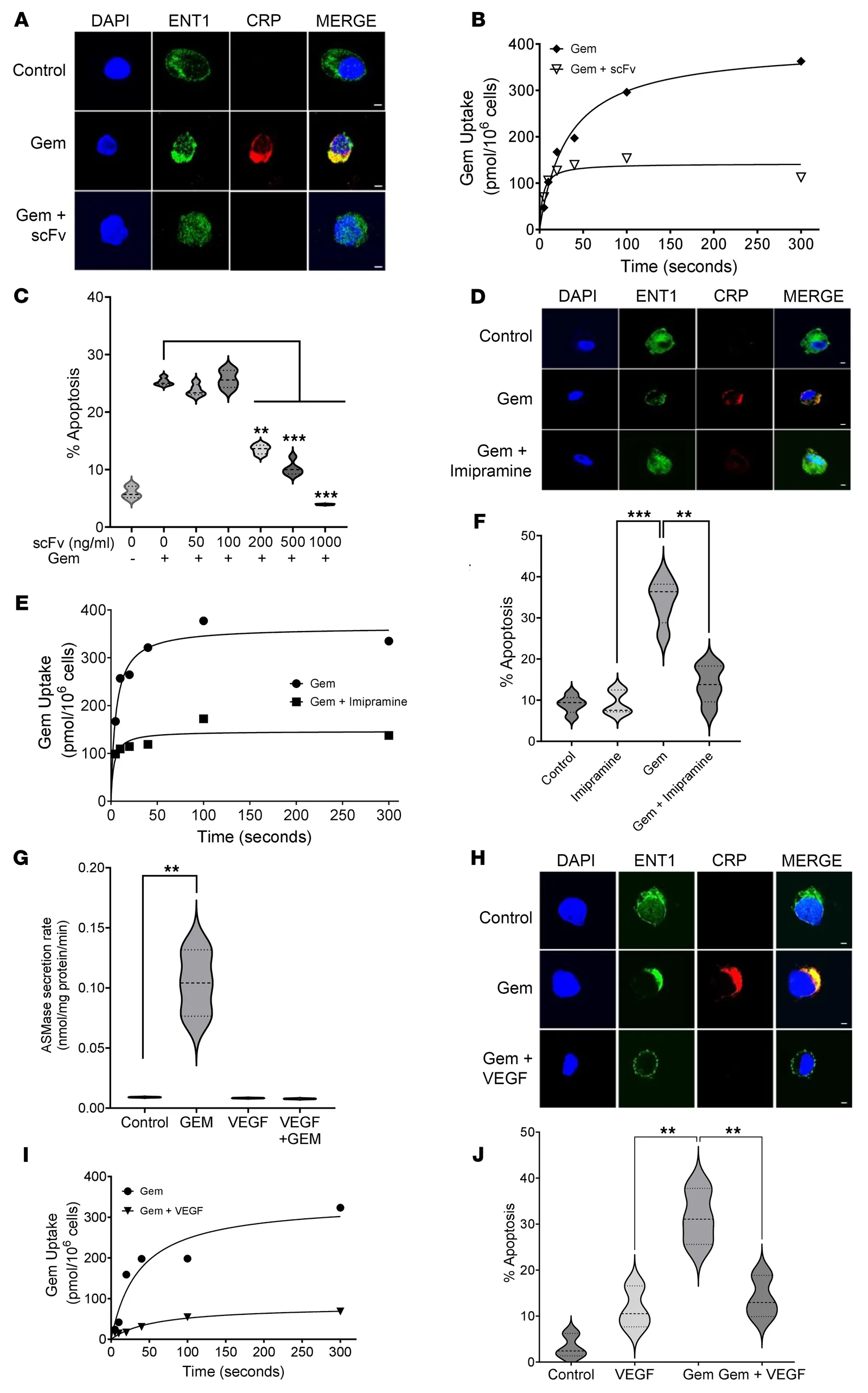
ASMase/ceramide signaling is obligate for ENT1 functionalization of gemcitabine uptake. (**A**) Confocal images showing colocalization of ENT1 in a CRP at 1 minute after 1 μM gemcitabine, inhibited by 1 µg/mL anti-ceramide 6B5 scFv. Proliferative BAECs were pretreated with scFv for 1 hour and imaged as in Figure 1B. Data are representative of 10 cells/group from 1 of 2 independent experiments. (**B**) Gemcitabine uptake in proliferative BAECs as in Figure 1A is inhibited by 1-hour pretreatment with 1 µg/mL 6B5 scFv. Data are representative of 3 independent experiments. (**C**) Dose-dependent scFv inhibition of apoptosis, quantified using bisbenzimide staining, at 16 hours after 10 µM gemcitabine treatment. Data (mean ± SEM) are collated from 3 independent experiments. ***P < 0.001, **P < 0.01 vs. gemcitabine treated alone by 1-way ANOVA. (**D**) Confocal images showing 50 µM imipramine inhibits ENT1 colocalization in CRPs at 1 minute after 1 µM gemcitabine. Proliferative BAECs were handled as in **A**. Data are representative of 10 cells/group from 1 of 2 independent experiments. (**E**) Gemcitabine uptake as in Figure 1A in the presence or absence of 50 μM imipramine. Proliferative BAECs were preincubated with imipramine for 30 minutes before 10 μM gemcitabine treatment. Data are representative of 3 independent experiments. (**F**) Imipramine (50 µM) pretreatment inhibits gemcitabine-induced proliferative BAEC apoptosis at 16 hours, quantified by bisbenzimide staining. Data represent mean ± SEM (*n* = 3). ***P < 0.001, **P < 0.01 by 1-way ANOVA. (**G**) VEGF (2 ng/mL) pretreatment of proliferative BAECs for 1 hour inhibits 10 µM gemcitabine-induced ASMase secretion measured at 1 minute. Data (mean ± SEM) were collated from 3 different experiments performed in triplicate. **P < 0.01 by 1-way ANOVA. (**H**) Confocal images showing VEGF (2 ng/mL) pretreatment for 1 hour inhibits ENT1 colocalization in CRPs at 1 minute after 1 µM gemcitabine. Cells were processed as described in **A**. (**I**) Uptake of gemcitabine in proliferative BAECs in the presence or absence of 2 ng/mL VEGF. Cells were handled as in **E**. Data are representative of 3 independent experiments. (**J**) VEGF (2 ng/mL) pretreatment inhibits gemcitabine-induced apoptosis at 16 hours, quantified by bisbenzimide staining. Data represent mean ± SEM (*n* = 3). ***P < 0.001 by 1-way ANOVA.

**Figure 3:**
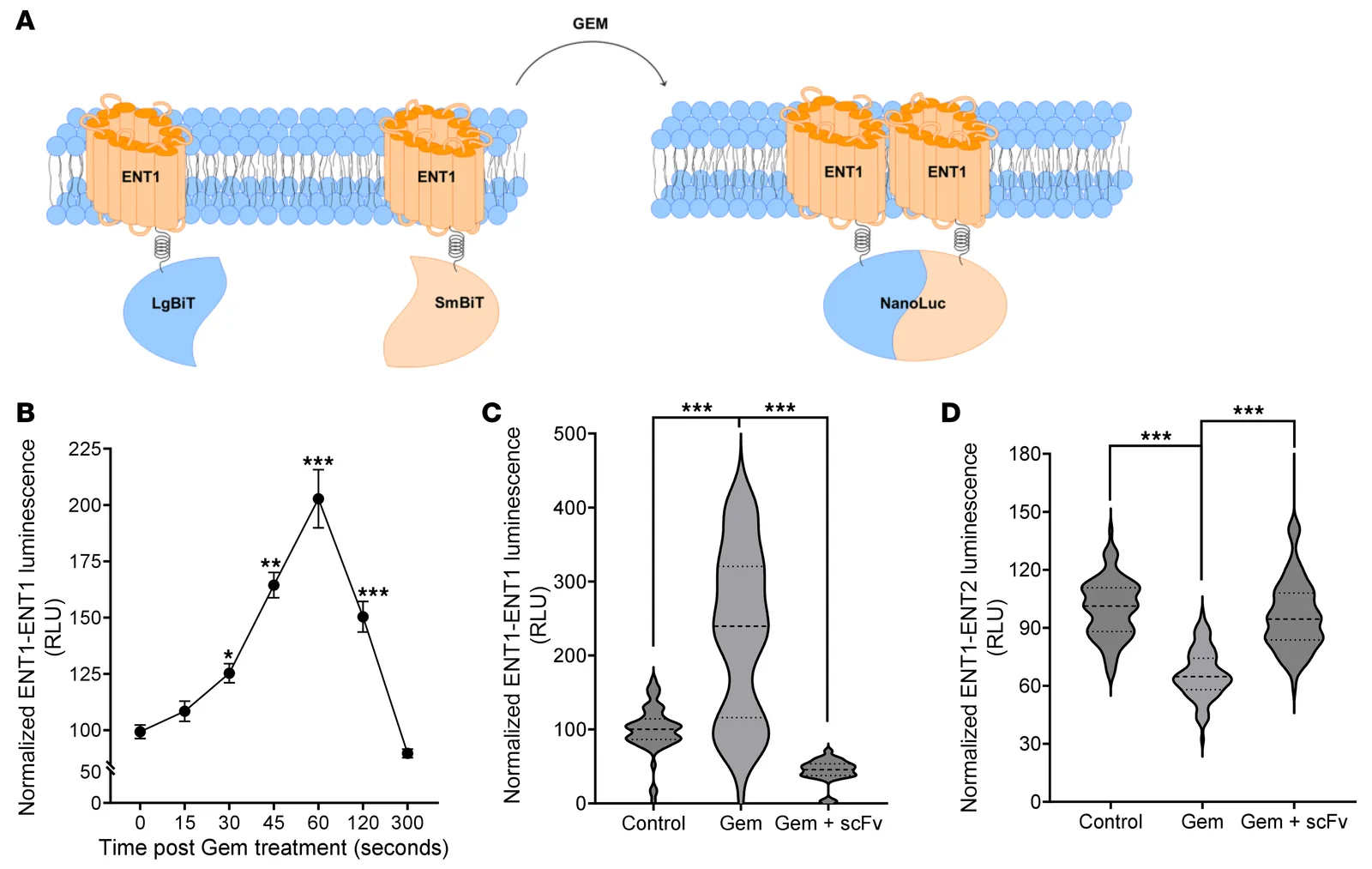
Gemcitabine induces ENT1 dimerization to drive gemcitabine uptake, an event inhibited by anti-ceramide scFv. (**A**) Illustrations of the NanoBiT technology used to monitor ENT1-ENT1 interaction. This image has been modified courtesy of the Promega Corp. Briefly, ENT1 fused to small BiT (SmBiT) is co-expressed in ENT-null HEK293 cells with either ENT1 or ENT2 fused to large BiT (LgBiT). ENT1 interaction with either ENT1 or ENT2, depending on experimental design, leads to structural complementation of LgBiT with SmBiT, generating a functional enzyme with a bright, luminescent signal. (**B**) HEK293 cells transiently cotransfected with ENT1–lgBiT and ENT1–smBiT plasmids were treated with gemcitabine (Gem) for different time points, and protein-protein interaction was measured as luminescence units (using SteadyGlo protocol from Promega) on a Glomax luminometer (Promega Corp.). Luminescence values were normalized to control luminescence values. ***P < 0.001, **P < 0.01, *P < 0.05 by 1-way ANOVA. (**C**) For scFv studies, HEK293 cells were pretreated with 1 μg/mL anti-ceramide 6B5 scFv for 15 minutes before gemcitabine treatment for 60 seconds in ENT1-LgBiT and ENT1–smBiT cotransfected cells. Data represent mean ± SEM (*n* = 60) from 1 of 2 independent studies. ***P < 0.001 by 1-way ANOVA. (**D**) HEK293 cells, transiently cotransfected with ENT2–lgBiT and ENT1–smBiT plasmids, were incubated for 15 minutes in the presence or absence of 1 μg/mL of anti-ceramide 6B5 scFv and then treated with gemcitabine for 1 minute. Protein-protein interaction was measured as in **B**. Data represent mean ± SEM (*n* = 96) from 1 of 2 independent studies. ***P < 0.001 by 1-way ANOVA.

**Figure 4:**
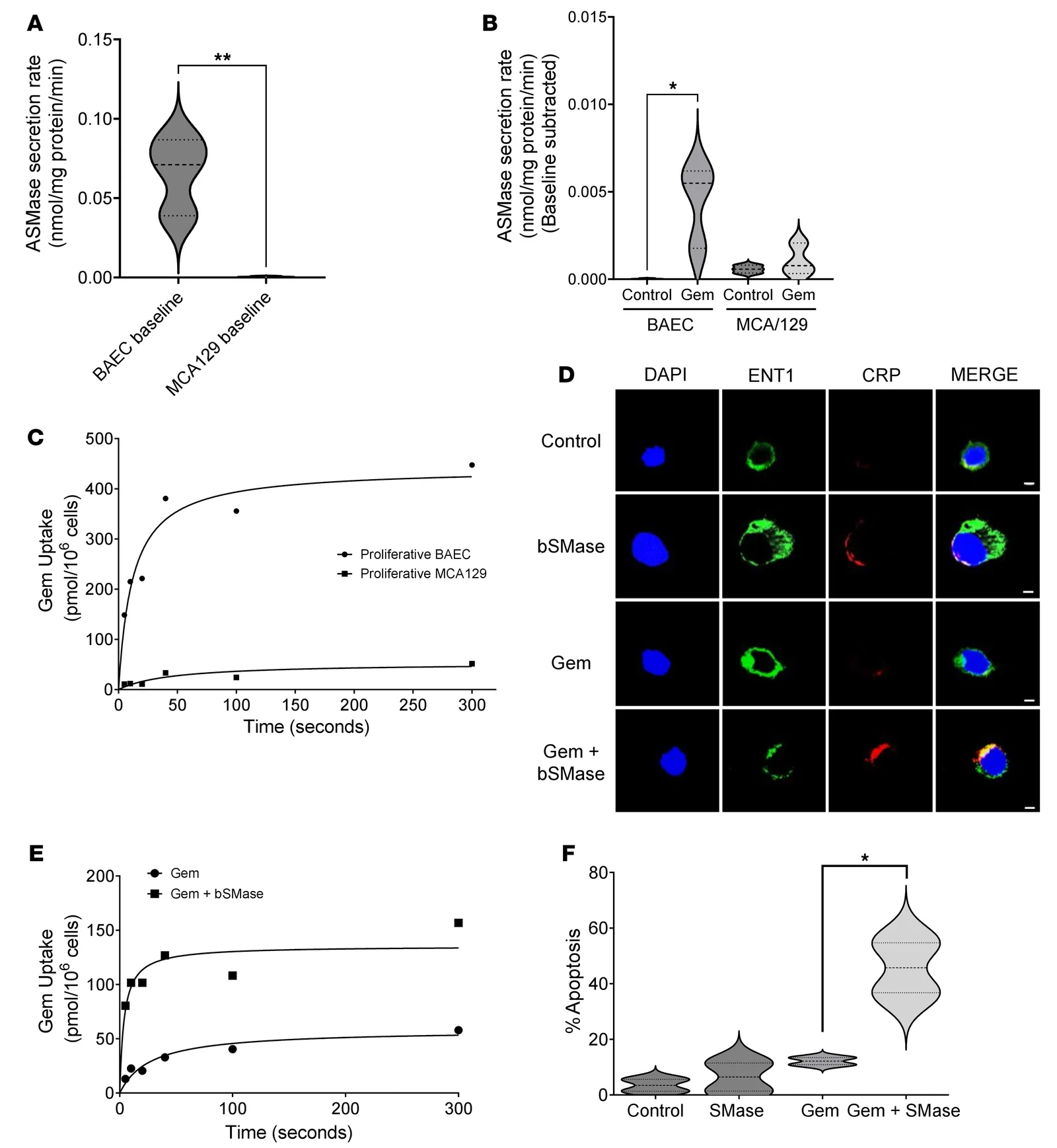
Exogenous bSMase confers ENT1 functionalization in gemcitabine-treated MCA/129 fibrosarcoma cells. (**A**) Baseline secreted ASMase activity was compared between proliferative BAECs and MCA/129 cells measured after 1 hour in serum-free media. Data (mean ± SEM) are collated from 3 different experiments performed in triplicate. **P < 0.01 by 2-tailed Student’s *t* test. (**B**) ASMase secretion rate in endothelial and MCA/129 cells (with baseline subtracted) treated with 10 μM gemcitabine (Gem) for 10 minutes. Data (mean ± SEM) were collated from 3 different experiments performed in triplicate. *P < 0.05 for GEM vs. control in BAECs by 1-way ANOVA. RLU, relative luminescence units. (**C**) Gemcitabine uptake as in Figure 1A in proliferative BAECs and MCA/129 cells. Data show representative results from 1 of 3 independent experiments. (**D**) bSMase confers co-localization of ENT1 within CRPs in proliferative MCA/129 cells at 1 minute after administration of 1 μM gemcitabine. Cells were pretreated with bSMase as in Figure 1G and stained as in Figure 1B. Data show 1 representative image of 10 individual cells in each group from 1 of 2 independent experiments. (**E**) Uptake of gemcitabine (10 μM gemcitabine plus tracer [^14^C] gemcitabine) in MCA/129 cells in the presence and absence of exogenous bSMase. Data show representative results from 1 of 3 independent experiments. (**F**) Pretreatment (1 hour) with bSMase (3 units/well) of proliferative MCA/129 cells confers gemcitabine-induced apoptosis at 16 hours (10 μM gemcitabine), assessed by bis-benzimide staining. Data (mean ± SEM) are collated from 2 experiments. *P < 0.05 vs. gemcitabine alone by 1-way ANOVA.

**Figure 5:**
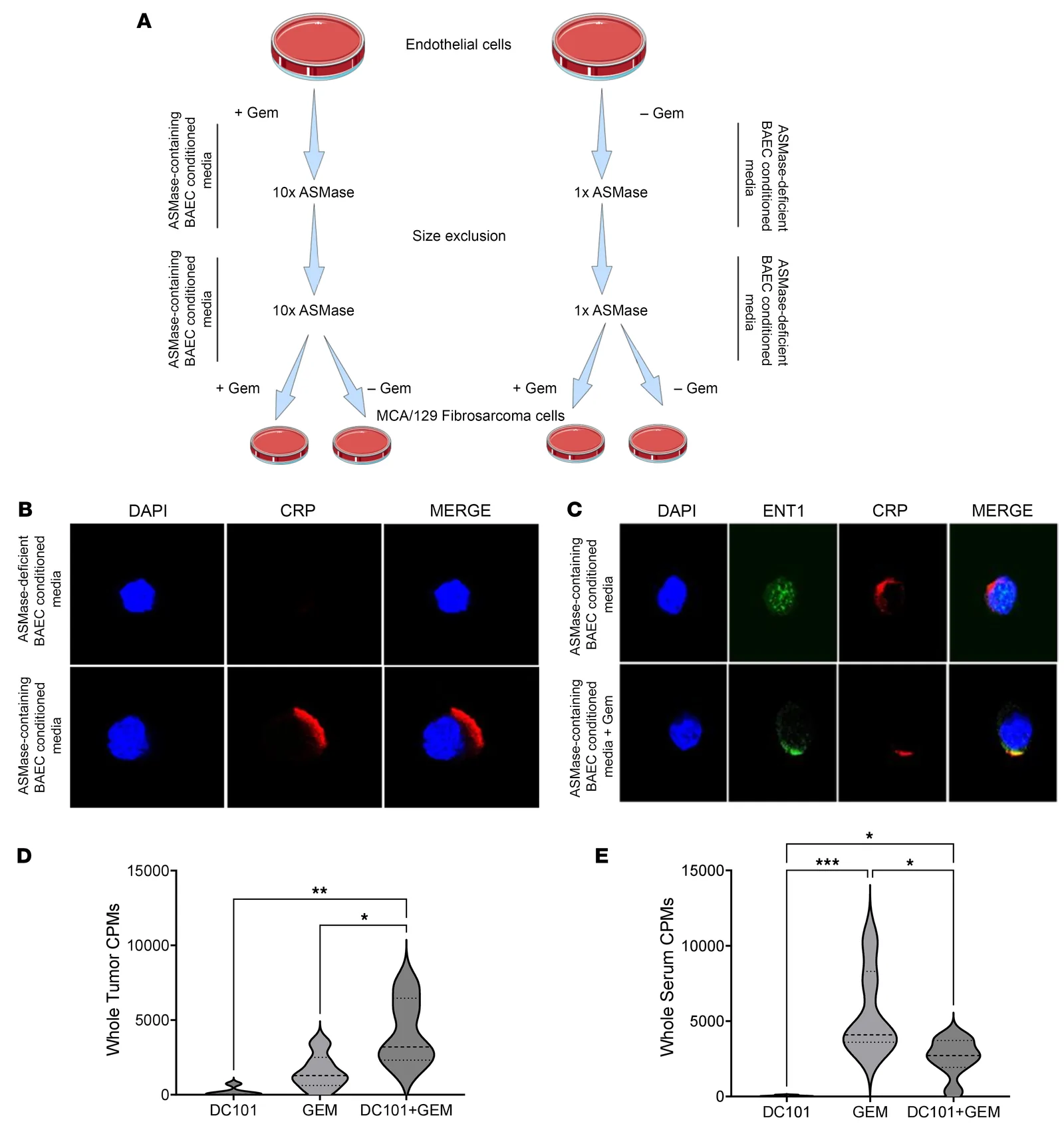
Secreted endothelial ASMase confers a “bystander” tumor response to gemcitabine. (**A**) Schema depicts step-by-step protocol followed for isolating conditioned medium from BAECs treated with gemcitabine (Gem), which was then used to confer bystander gemcitabine-induced activation of MCA/129 fibrosarcoma cells. (**B** and **C**) Conditioned medium from gemcitabine-treated, but not untreated, BAECs containing secreted ASMase, confers formation of CRPs (**B**) onto MCA/129 fibrosarcoma cells into which (**C**) ENT1 inserts (at 1 minute). Media from BAEC treated with or without 10 μM gemcitabine for 10 minutes were collected and subjected to size exclusion chromatography by Amicon Ultra-4 centrifuge filters (molecular cutoff, 30 KDa) to remove gemcitabine. Thereafter, proliferative MCA/129 cells were exposed to conditioned medium for 1 minute and prepared for CRP detection as in Figure 1B. Data show 1 representative of 10 individual cell images per group from 1 of 2 independent experiments in **B** and **C**. (**D**) DC101 (1.6 mg/mouse, i.v.) enhances gemcitabine (240 mg/kg “cold” gemcitabine plus ^3^H-gemcitabine “tracer” i.p.) uptake into MCA/129 fibrosarcoma flank tumors. Briefly, 2 × 10^6^ MCA/129 cells were s.c. implanted into the flanks of athymic nude mice (Envigo), and mice harboring 100–150 mm^3^ tumors were treated with DC101 at 1 hour before gemcitabine treatment. At 1 hour after gemcitabine administration, mice were sacrificed, tumors were collected and solubilized using Solvable Tissue Solubilizer (PerkinElmer, 6NE9100) and hydrogen peroxide, and tumor-associated ^3^H-gemcitabine was quantified by liquid scintillation counting. (**E**) Radiolabeled gemcitabine counts in whole-blood serum of mice sacrificed from **D**. Data (mean ± SEM) in **D** and **E** were derived from 5 mice/group. *P < 0.05, **P < 0.01, ***P < 0.001 by 1-way ANOVA.

**Figure 6:**
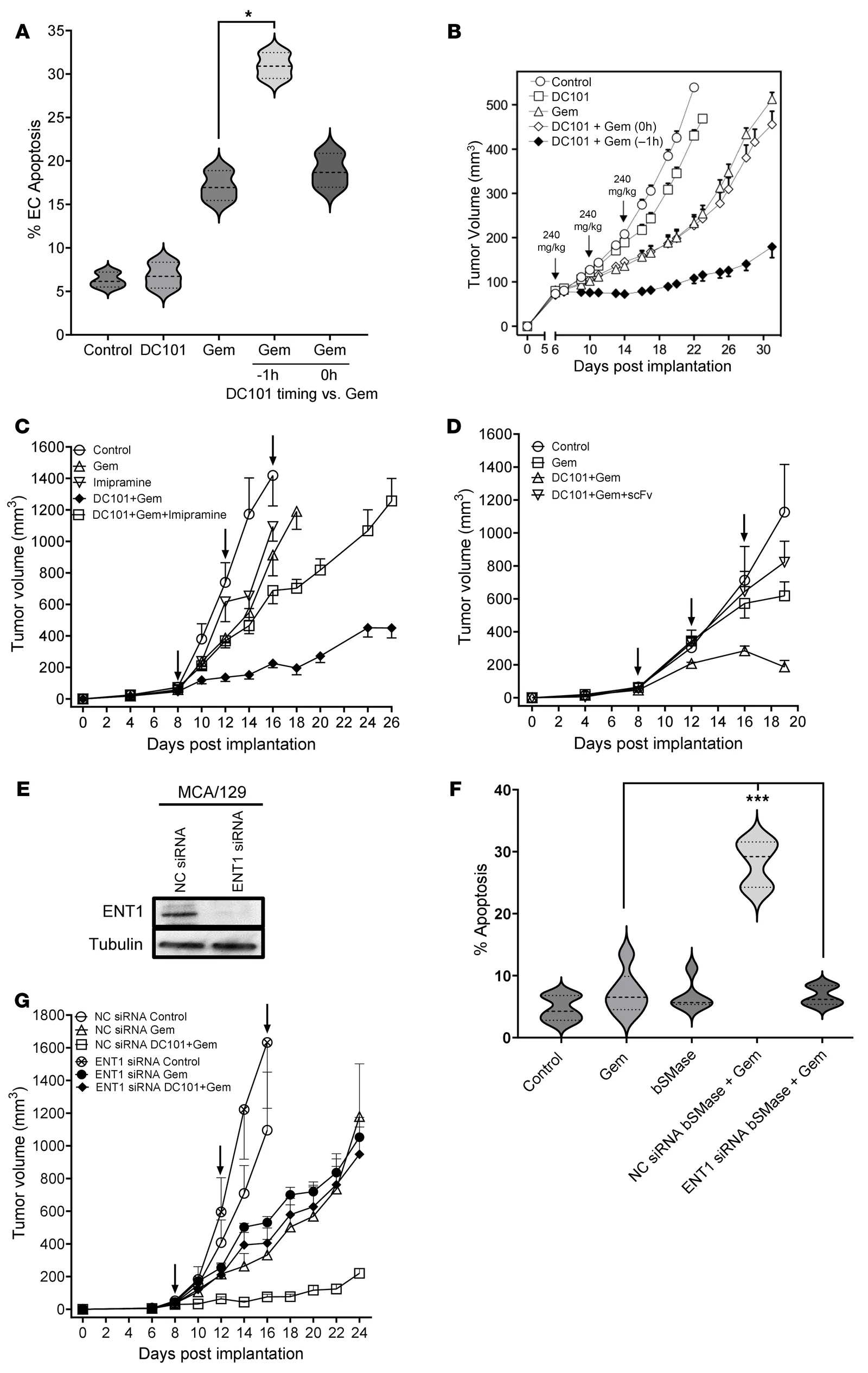
Synchronized timing of antiangiogenic drug to gemcitabine confers enhanced ASMase- and ceramide-dependent MCA/129 sarcoma response. (**A** and **B**) Impact of timing of antiangiogenic drug delivery relative to gemcitabine (Gem) on the endothelial apoptotic response (**A**) and tumor volume (**B**) of MCA/129 tumors implanted in the flanks of sv129/BL6 mice. DC101 (1.6 mg/25 g mouse i.v.) was delivered either 1 hour prior to or immediately before gemcitabine exposure (240 mg/kg i.p.). At 4 hours after treatment, tumors were harvested and endothelial cell apoptosis quantified by co-staining for TUNEL and the endothelial-specific mAb MECA-32, as previously published (40, 66). Tumor volume was measured as in Methods. Data (mean ± 95% CI) were compiled from 20 different fields (×400 magnification) from 2 tumors at each time point (**A**). *P < 0.05 by 1-way ANOVA. Data (mean ± SEM) are collated from 5 mice/group (**B**). *P* < 0.001 for DC101 plus gem (0 h) vs. DC101 plus gem (−1 h). (**C**) MCA/129 cells were implanted in the flanks of athymic nude mice (Envigo). At a volume of 50–70 mm^3^, tumor-bearing mice were treated with imipramine (10 mg/kg, twice daily) on the day preceding and the day of gemcitabine treatment, for a total of 3 doses. DC101 (1.6 mg/25 g mouse, i.v.) was administered 1 hour before gemcitabine treatment (240 mg/kg, i.p.). *n* = 4–5 mice/group. (**D**) MCA/129 cells were implanted and mice were treated as described in **C**. In the scFv group, animals received anti-ceramide 6B5 scFv (150 μg/mouse) 10 minutes before DC101 administration. Gemcitabine (240 mg/kg, i.p.) was administered 1 hour after DC101 treatment (1.6 mg/25 g mouse, i.v.). *n* = 4–5 mice/group. (**E**) Western blot analysis of ENT1 protein levels in MCA/129 cells transfected with nontargeting control (NC) or ENT1 siRNA using Lipofectamine 3000 following the manufacturer’s instruction. Cell lysates were collected 48 hours after transfection. (**F**) MCA/129 cells transfected with NC or ENT1 siRNA were treated with gemcitabine in the presence or absence of bSMase (3 units/well). Apoptosis was quantified using bis-benzimide at 16 hours after treatment. Data represent mean ± SEM (*n* = 3). ***P < 0.001 by 1-way ANOVA. (**G**) MCA/129 cells transfected with NC or ENT1 siRNA were implanted and mice were treated as described in **C**. DC101 (1.6 mg/25 g mouse, i.v.) was administered either 1 hour before or immediately before gemcitabine treatment (240 mg/kg, i.p.). *n* = 5 mice per group.

**Figure 7:**
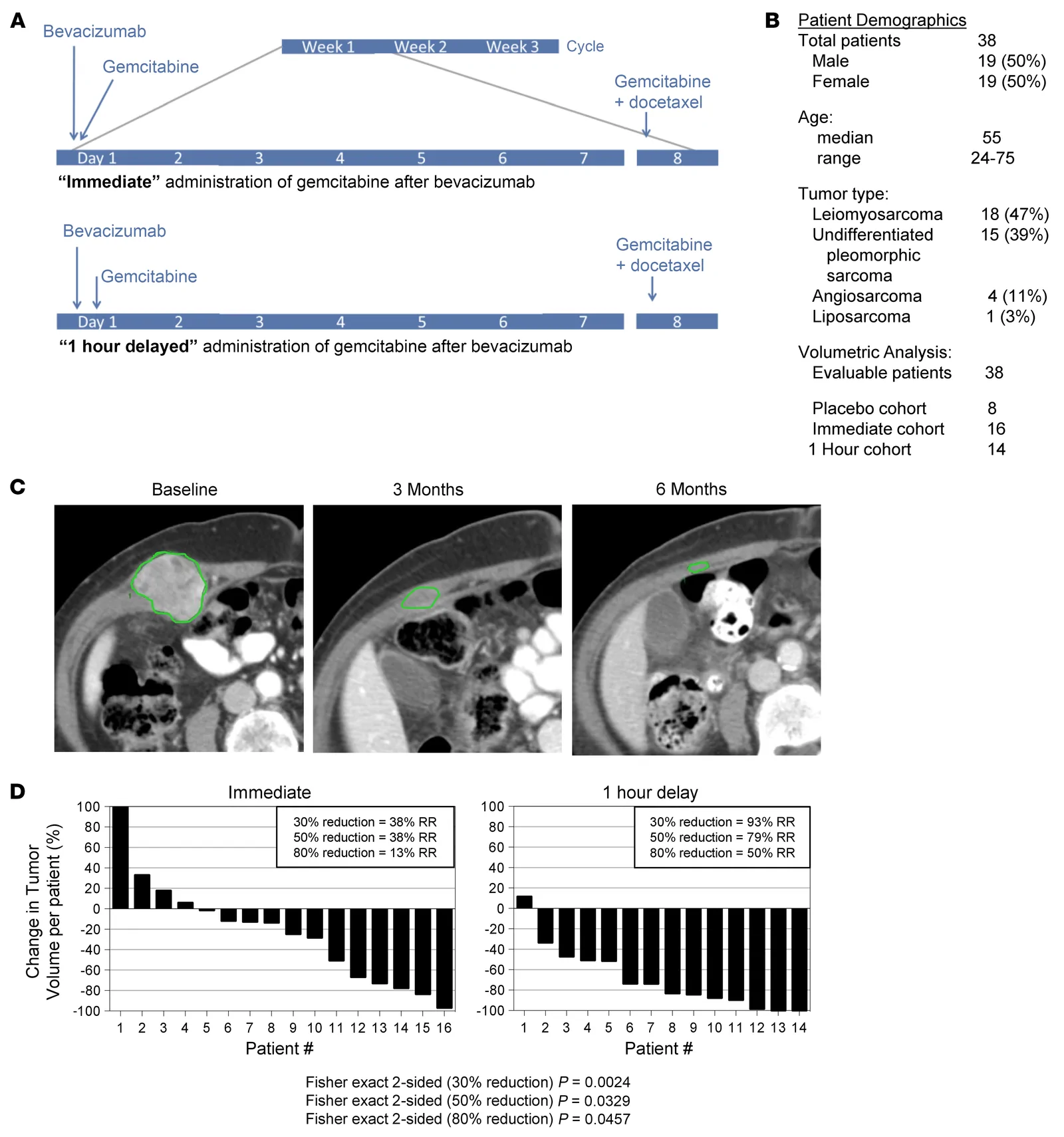
Optimized timing of antiangiogenic therapy with gemcitabine correlates with improved patient outcomes in a phase II advanced sarcoma trial. (**A**) Schematic representation of the original prospective phase II study of gemcitabine and docetaxel with bevacizumab in advanced sarcoma (upper panel), which was subsequently converted to a single-arm, open-label, nonrandomized study (lower panel). (**B**) Demographics of patients with metastatic sarcoma treated with bevacizumab, gemcitabine, and docetaxel on the prospective phase II study at MSKCC. (**C**) Tumor volumes were measured by a radiologist, who was blinded to the treatment timing, to determine the nadir volume during the study using serial follow-up imaging. Shown is a representative CT time course of an abdominal wall metastasis at baseline (left), 3 months (center), and 6 months (right) in patient 8 in the 1-hour-interval bevacizumab cohort. Tumor contour is outlined in green. (**D**) Waterfall plots demonstrating best tumor response based on volumetric change for each patient treated with placebo or bevacizumab immediately before (left) or 1 hour before (right) gemcitabine. RR, response rate.

## Version 1 09/03/2026

In-Press Preview

## Version 2 10/15/2026

Electronic publication
