# Supplementary material for: Enhancing gemcitabine uptake by promoting dimerization of ENT1 transporter improves response rates of patients with sarcoma
Source: J Clin Invest. 2026 Sep 3;136(20):e183474. doi: 10.1172/JCI183474 (PMC13668608; doi:10.1172/JCI183474)
Supplement: Supplementary file 1 [file jci-136-183474-s246.pdf]

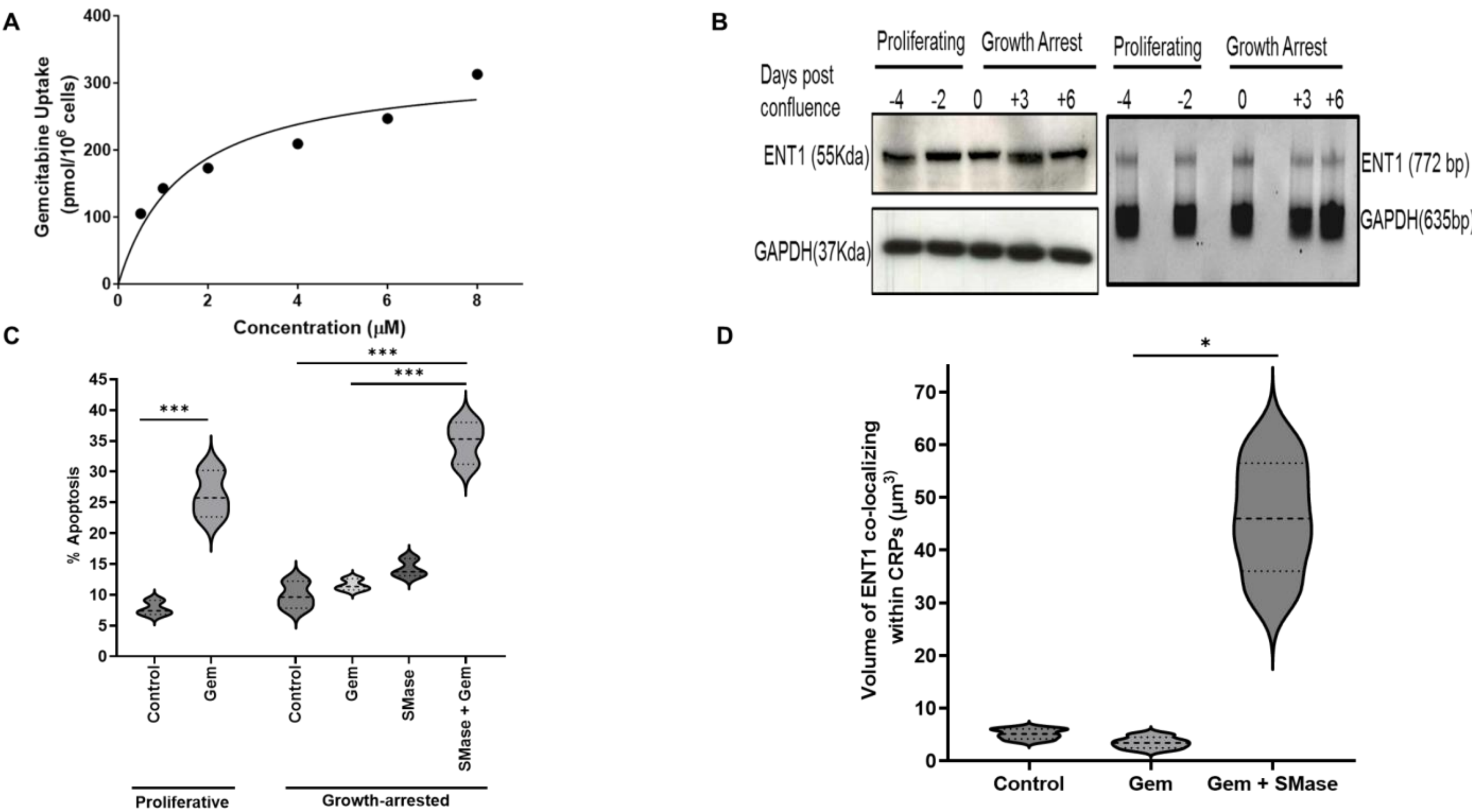

Supplemental Figure 1: Kinetics of gemcitabine uptake and expression of ENT1 in BAECs. A. Michaelis-Menten Kinetics analysis of gemcitabine uptake at 1 min in proliferative BAECs. Data represent best fit of 1 of 3 independent experiments. **B.** Left – ENT1 protein levels in proliferative and growth-arrested BAEC. Right – ENT1 mRNA levels in proliferative and growth-arrested BAEC. **C.** Apoptosis was quantified in proliferative and growth-arrested cells using bis-benzimide assay. Proliferative cells were treated with gemcitabine (10 μM) whereas the growth-arrested cells were treated with bSMase (5 units) or gemcitabine alone or together. ***$p<0.001$ by 1-way ANOVA. **D.** Volume of ENT1 colocalizing with CRPs in growth-arrested cells pretreated with bSMase prior to 1 min of 1 μM gemcitabine treatment. Data (Mean±SEM) are collated from two independent experiments performed in triplicate. *$p<0.05$ by 1-way ANOVA.

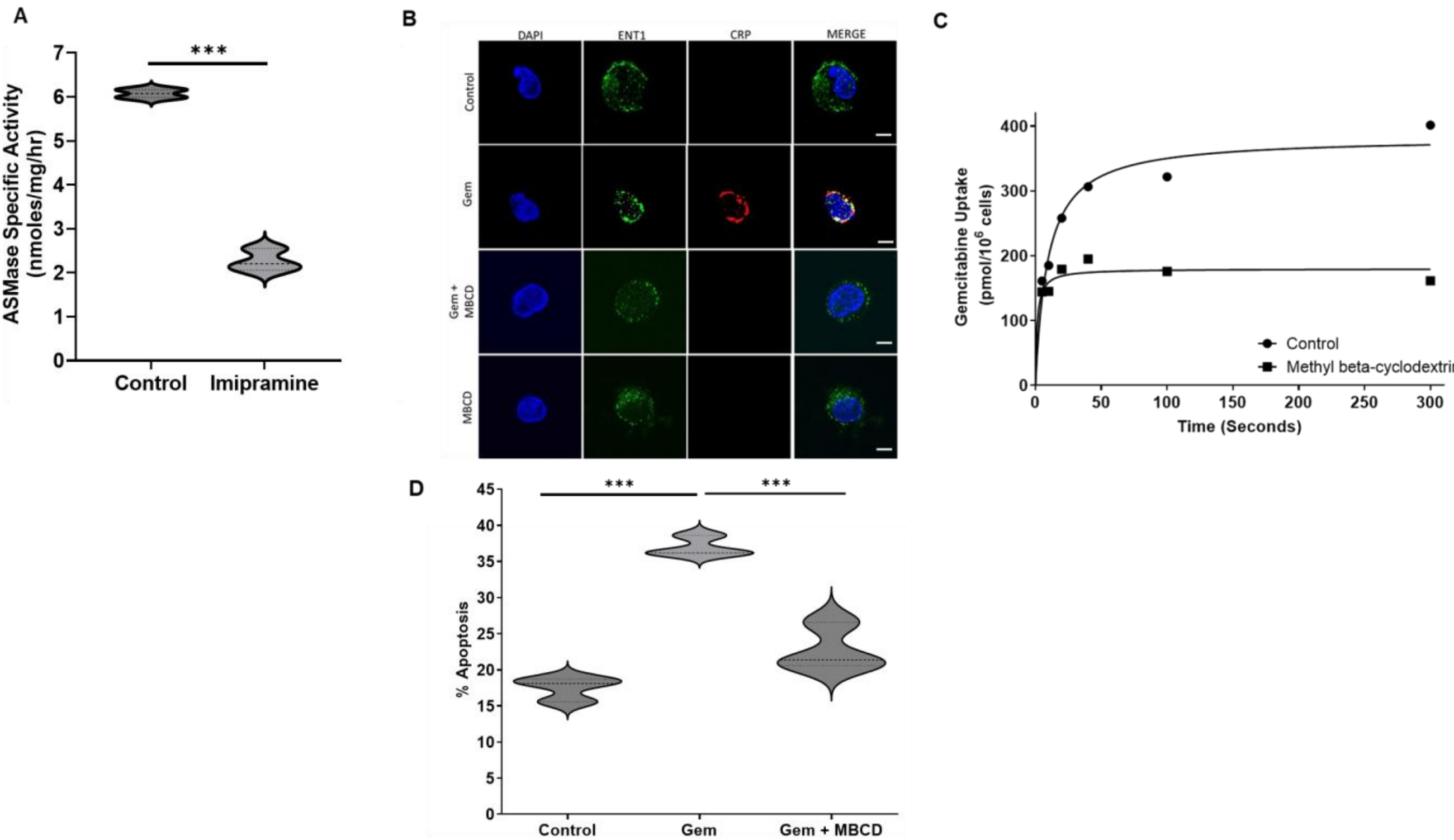

Supplemental Figure 2: Imipramine and methyl beta-cyclodextrin (MBCD) inhibit gemcitabine uptake by repressing ASMase activity. **A.** Proliferative BAECs were treated with 50 μM imipramine for 30 min and total cellular ASMase activity was determined. Data (Mean ± SEM) are collated from 3 independent experiments. ***p<0.001 by 2-tailed Student's *t* test. **B.** Confocal images showing co-localization of ENT1 in a CRP in cells pretreated with 5 mM MBCD before gemcitabine treatment. **C.** Gemcitabine uptake in proliferative cells pretreated with 5 mM MBCD. **D.** MBCD-mediated inhibition of gemcitabine (10 μM) induced apoptosis in proliferative BAECs. Cells were treated with 5mM MBCD 1 hour prior to gemcitabine treatment. Cells were harvested after 16 hours, and apoptosis was quantified using bis-benzimide staining. Data (Mean ± SEM) are collated from 3 independent experiments. ***p<0.001 by 1-way ANOVA.

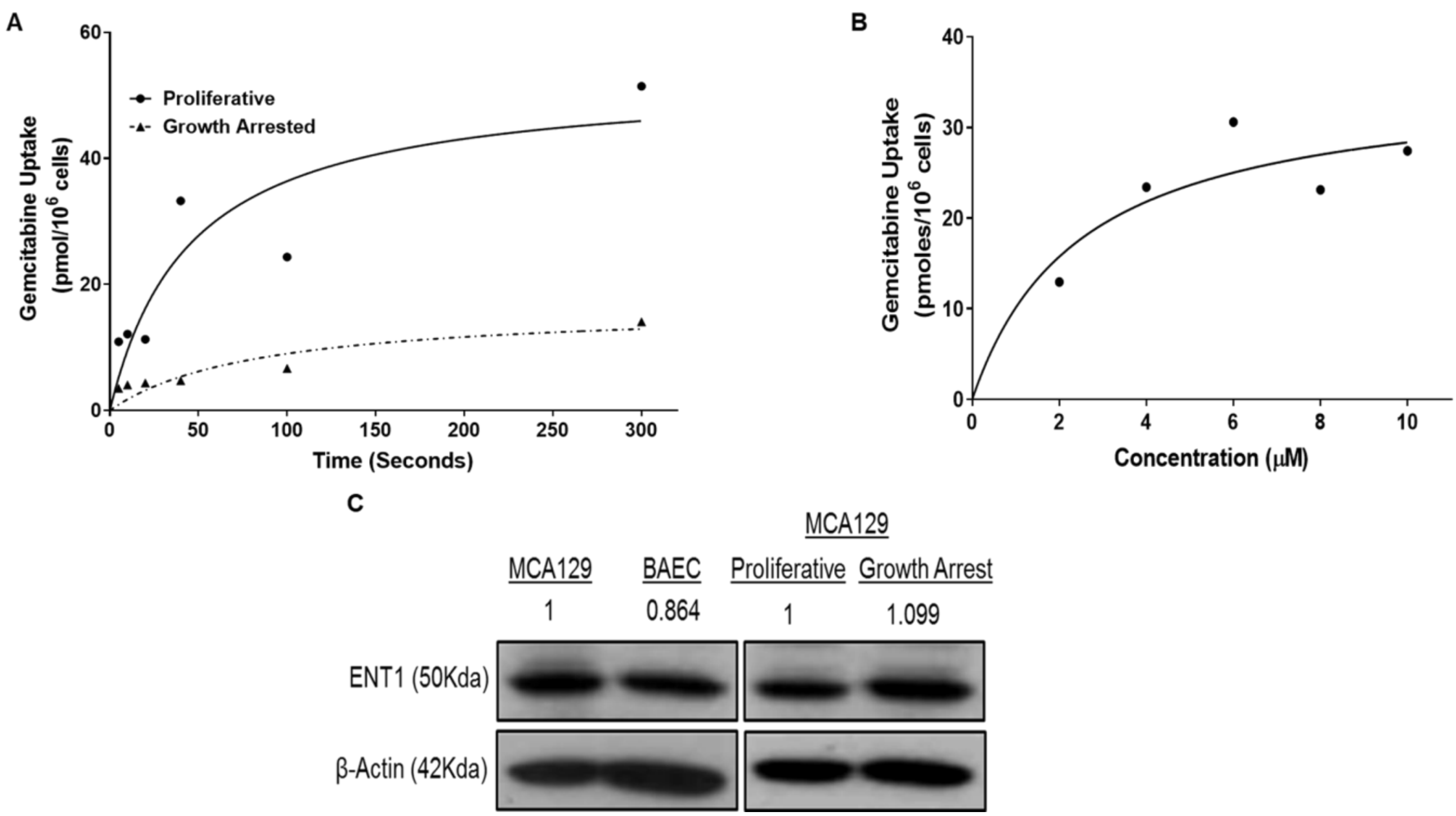

Supplemental Figure 3: Kinetics of gemcitabine uptake and expression of ENT1 in MCA/129 fibrosarcoma cells. **A.** Uptake of gemcitabine (10 μM gemcitabine + tracer [^{14}C]gemcitabine) in proliferative and growth-arrested MCA/129 cells. **B.** Michaelis-Menten kinetic analysis of gemcitabine uptake at 1 min in proliferative MCA/129 cells. Data represent best fit of data from 1 of 3 independent experiments. **C.** Left – Comparison of protein expression of ENT1 in BAECs and MCA/129 cells. Right – Comparison of ENT1 protein expression between proliferative and growth-arrested MCA/129 cells. Numbers represent the densitometry of ENT1 with respect to β-Actin. Data represent 1 of 2 independent experiments.

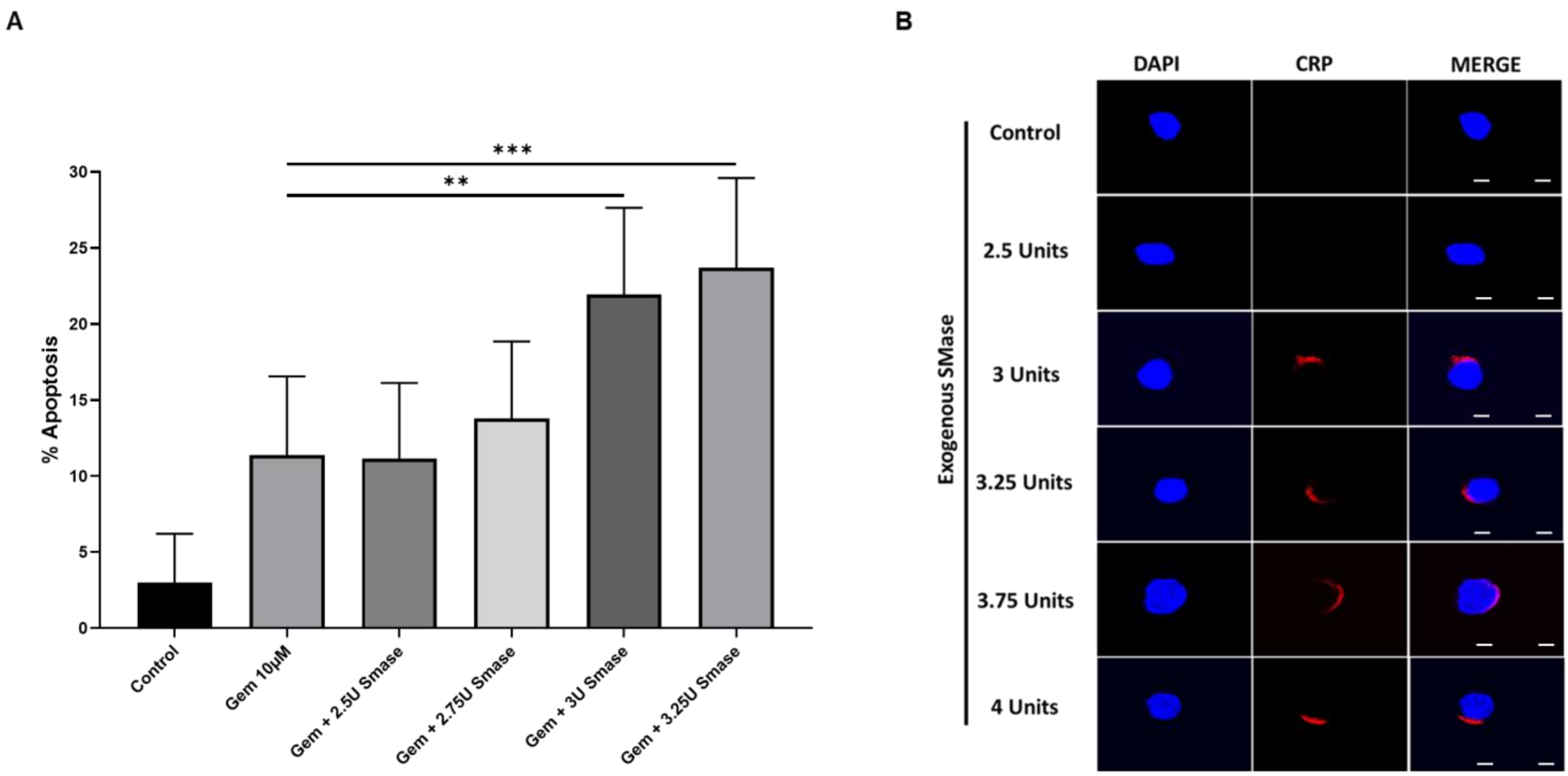

Supplemental Figure 4: Exogenous sphingomyelinase rapidly restores gemcitabine-induced apoptosis in proliferative MCA/129 cells. **A.** Bacterial sphingomyelinase (*Bacillus cereus;* bSMase; 2.5-3.25 units) was applied with gemcitabine to proliferative MCA/129 cells and apoptosis was quantified using bis-benzimide assay after 16 hours. Error bars represents 95% confidence interval. *p<0.05, **p<0.01, ***p<0.001 vs. gemcitabine alone by two-tailed Student's t test. **B.** Dose-dependent bSMase-induced CRP formation in MCA/129 cells at 1 min of stimulation. The cell nucleus is stained with DAPI (blue signal), CRPs are stained with mouse anti-ceramide MID 15B4 (1:50 dil) followed by Cy3-conjugated anti-mouse IgM (red signal). Data are representative of 1 of 2 independent experiments.

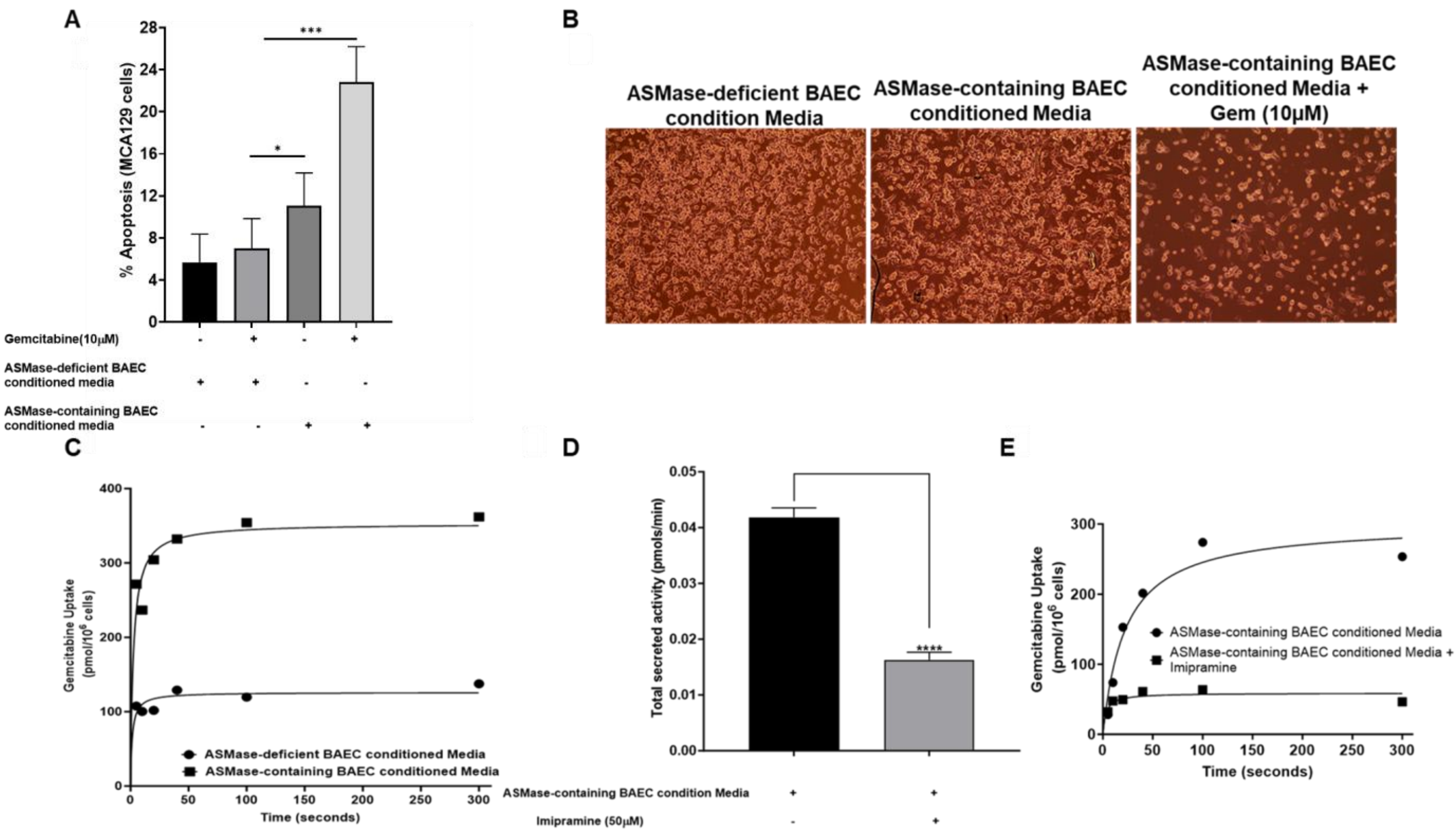

Supplemental Figure 5: BAEC conditioned media plus gemcitabine induces apoptosis in MCA/129 cells. **A.** BAECs were treated with gemcitabine (10 μM) for 10 min and conditioned media was collected and concentrated using Amicon Ultra-4 centrifuge filters (molecular cutoff 30kDa). Concentrated media was then exposed to MCA/129 cells and apoptosis was quantified using bis-benzimide assay at 16 hours. Data (Mean ± SEM) are collated from 3 independent experiments. $^*p<0.05$, $^{***}p<0.001$ vs. ASMase-deficient BAEC conditioned media + gem by two-tailed Student's t test. **B.** Representative pictures of ASMase-deficient BAEC conditioned media, ASMase-containing BAEC conditioned media and ASMase-containing BAEC conditioned media plus gemcitabine re-addition. **C.** Gemcitabine uptake in MCA/129 fibrosarcoma cells treated with ASMase-deficient and ASMase-containing BAEC conditioned media. **D.** Total secreted ASMase activity in ASMase-containing BAEC conditioned media pretreated with imipramine (50 μM). $^{***}p<0.001$ by two-tailed Student's t test. **E.** Gemcitabine uptake in MCA/129 cells incubated with ASMase-containing BAEC conditioned media pre-treated with imipramine. Data show representative results from one of three independent experiments.

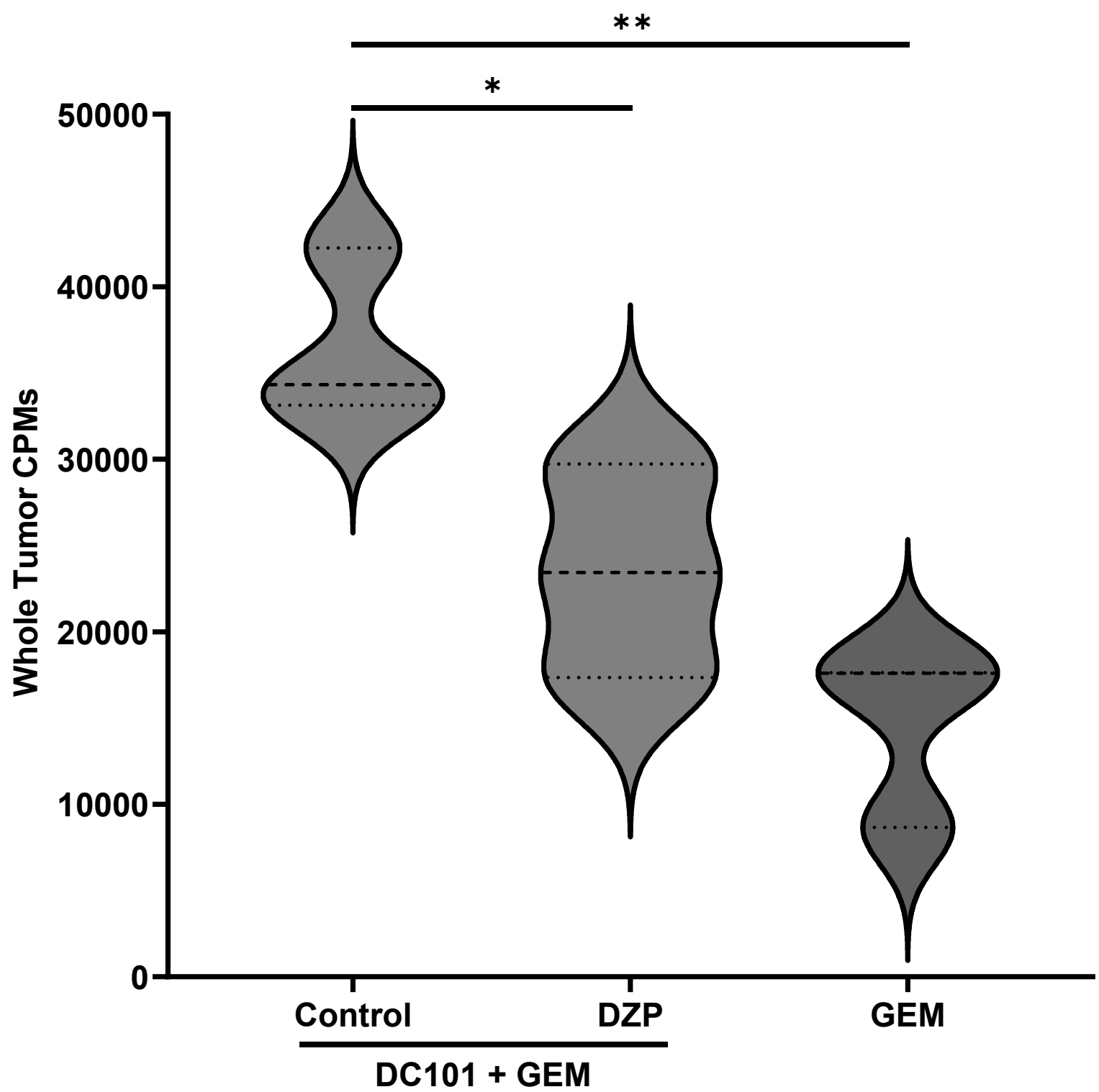

Supplemental Figure 6: ENT1 inhibitor Dilazep inhibits DC101-mediated gemcitabine uptake in MCA/129 fibrosarcoma tumors. The specific ENT1 inhibitor Dilazep (DZP) prevents gemcitabine uptake in MCA/129 fibrosarcoma flank tumors. Briefly, 240 mg/kg "cold" gemcitabine + ^{3}H-gemcitabine as "tracer", i.p. given 1 hour after DC101 (as in Fig. 5D) were administered in the nude mice with or without 20 mg/kg DZP i.p. A second hydrophobic ENT1 inhibitor, NBMPR, could not be formulated for delivery in aqueous suspension despite extensive exploration. For these studies, MCA/129 tumors were handled as in Fig. 5D**.** *$p<0.05$, **$p<0.01$ by 1-way ANOVA.

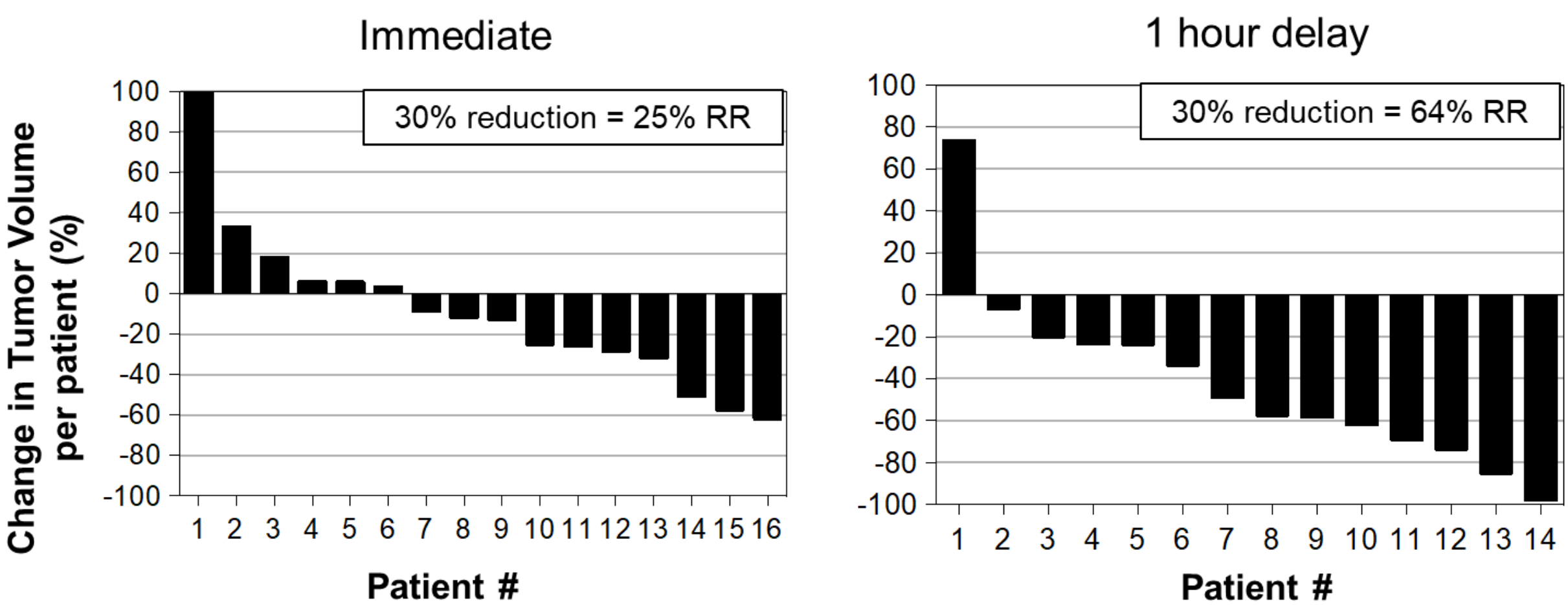

1-sided Wilcoxon rank-sum (Mann-Whitney) test: p=0.0075
1-sided 2-sample proportion test p=0.0362

Supplemental Figure 7: Delivery of bevacizumab at 1 hour prior to gemcitabine improves tumor response in patients with sarcoma at the time of the first scan after initiating treatment (after two 3-week treatment cycles). Patients with metastatic sarcoma on a phase II study at MSKCC were evaluated as in Fig. 7. Waterfall plots quantify tumor response at the initial imaging assessment based on volumetric change for each patient treated with bevacizumab immediately before (left), or 1 hour before (right), gemcitabine.

**Pancreatic Cancer**	Ewing Sarcoma
**Non-Small Cell Lung Cancer**	Osteosarcoma
**Breast Cancer**	Head and Neck Cancer
**Ovarian Cancer**	Small Cell Lung Cancer
Angiosarcoma	Peripheral T-Cell Lymphoma
Dedifferentiated Chordoma	Hodgkin Lymphoma
Marginal Zone Lymphomas	Uterine Sarcoma
Bladder Cancer	Gall Bladder and Bile Duct Cancer

Supplemental Table 1: List of medically approved (in bold) and medically accepted indications for gemcitabine use in the treatment of cancer. Information is based on NCCN Clinical Practice Guidelines in Oncology published by the National Comprehensive Cancer Network.

Nucleoside drugs for anti-cancer therapy	Nucleoside drugs for anti-viral therapy
Gemcitabine	Remdesivir
Capecitabine	Abacavir
Cytarabine (Ara-C)	Acyclovir
Trifluridine	Zidovudine
Tegafur	Stavudine
Doxifluridine	Cidofovir
Azacytidine	Famciclovir
Cladribine	Entecavir
Fludarabine	Valaciclovir
Floxuridine	Ribavirin

Supplemental Table 2: List of FDA-approved nucleoside-based anti-cancer and anti-viral drugs

Supplemental Movie 1: 3D rendering of CRP formation in Control BAECs

Supplemental Movie 2: 3D rendering of CRP formation in gemcitabine-stimulated BAECs
