# Supplementary material for: Enhancing gemcitabine uptake by promoting dimerization of ENT1 transporter improves response rates of patients with sarcoma
Source: J Clin Invest. 2026 Sep 3;136(20):e183474. doi: 10.1172/JCI183474 (PMC13668608; doi:10.1172/JCI183474)

Fig. 6 E

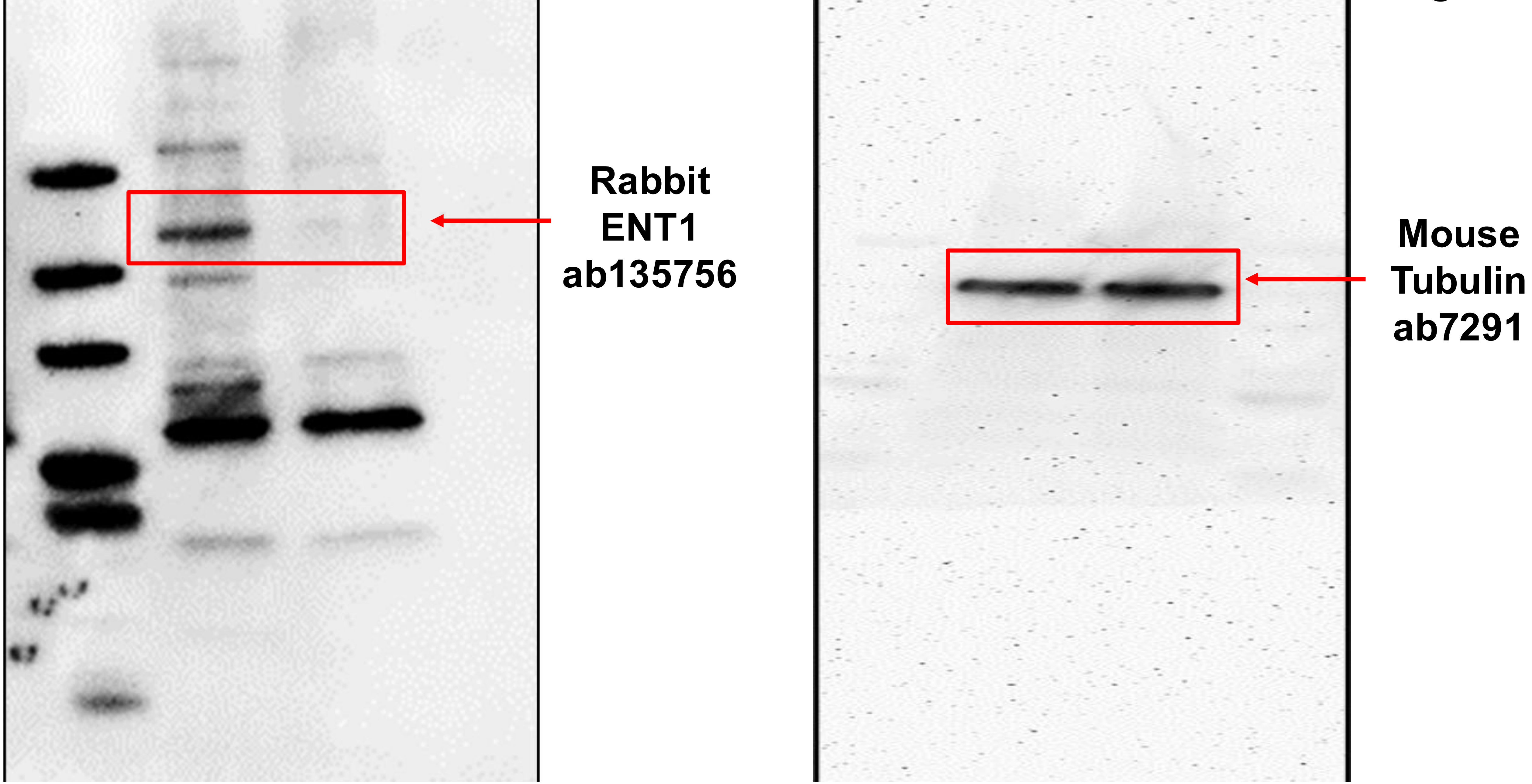

Supplemental Fig. 1B

Semi Quantitative PCR 8-31-2017

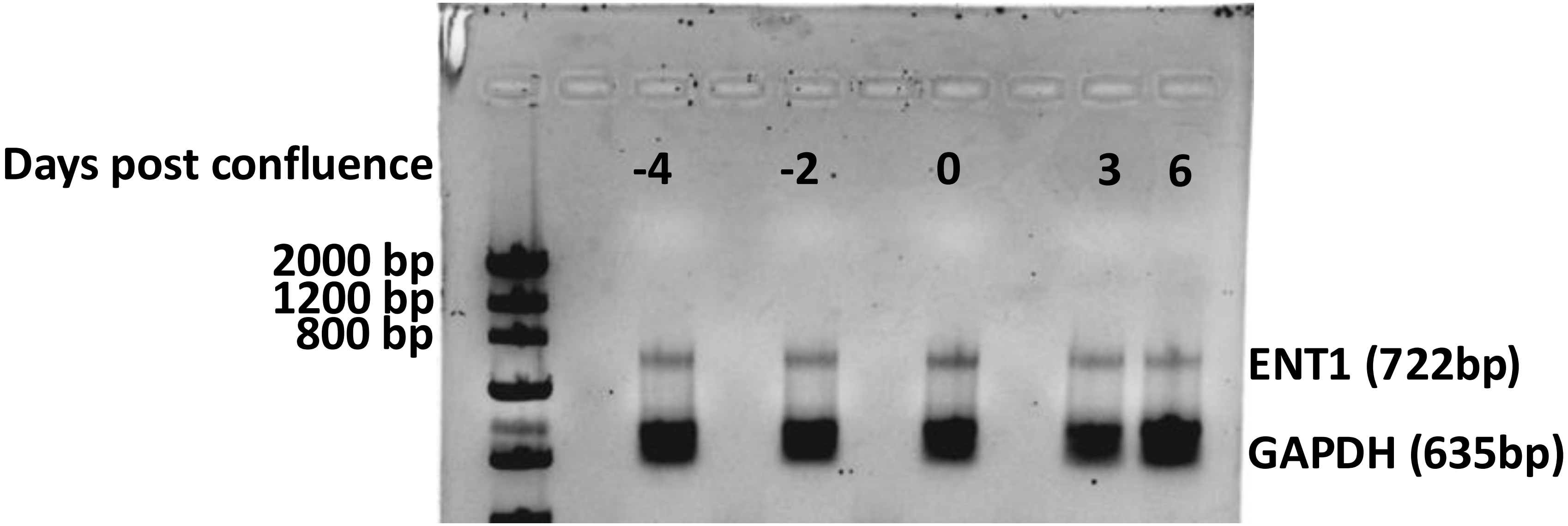

Supplemental Fig. 1B

Days post confluence -4 -2 0 +3 +6

ENT1 (55Kda)

GAPDH(37Kda)

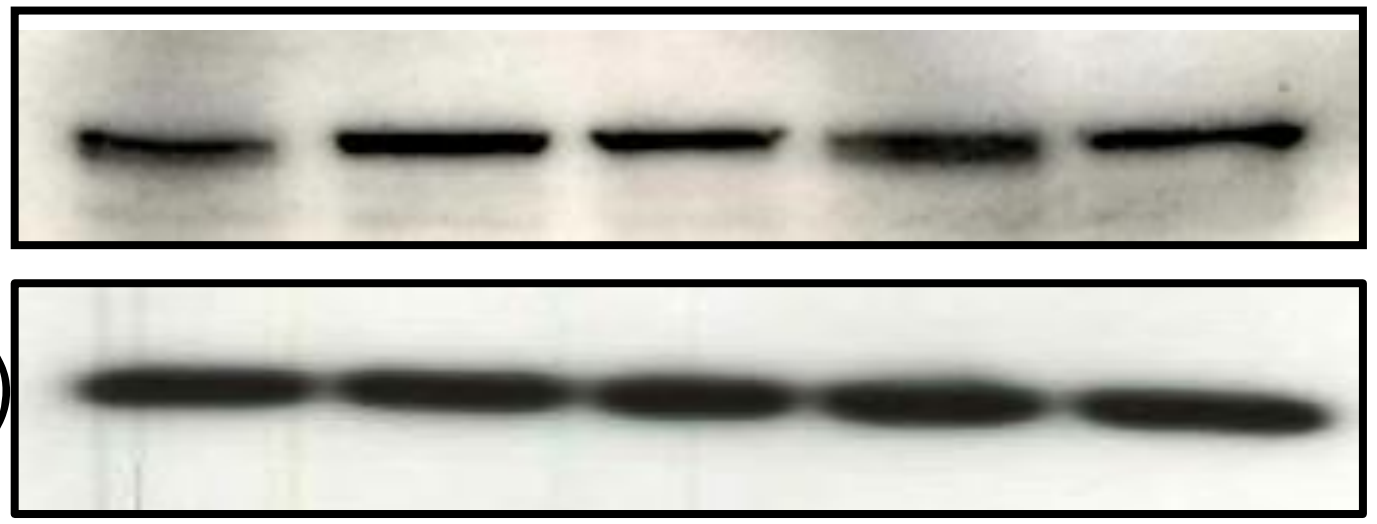

ENT1 Raw Data 9-08-2017

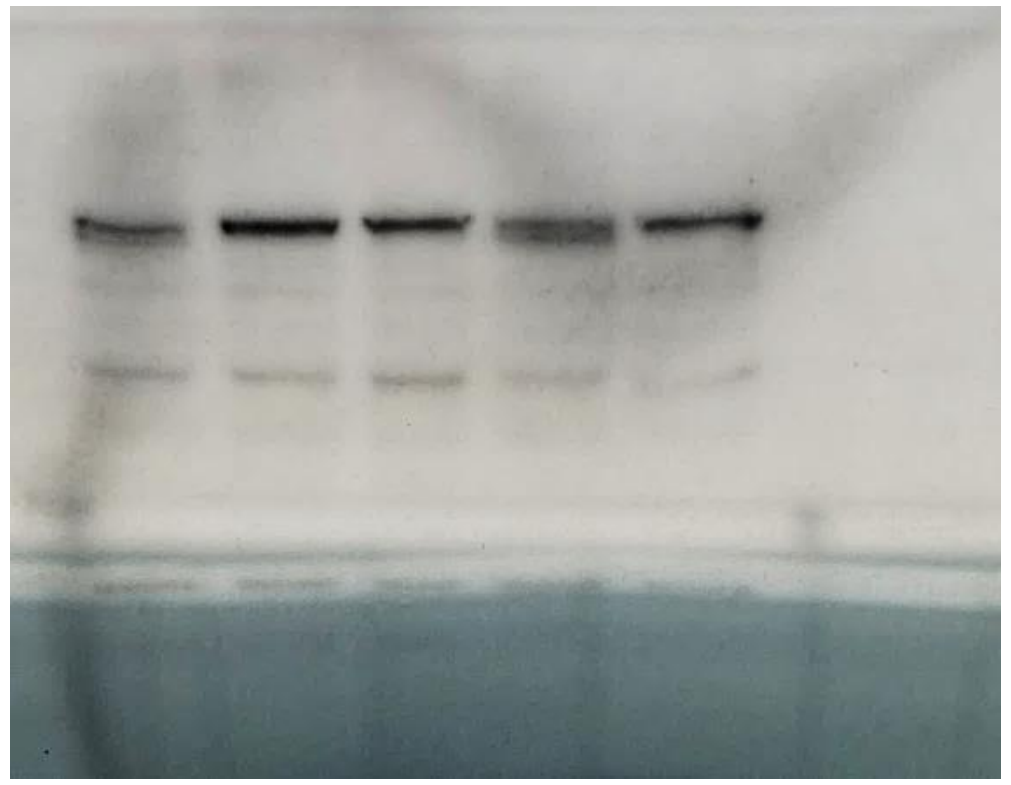

GAPDH Raw Data 9-9-2017

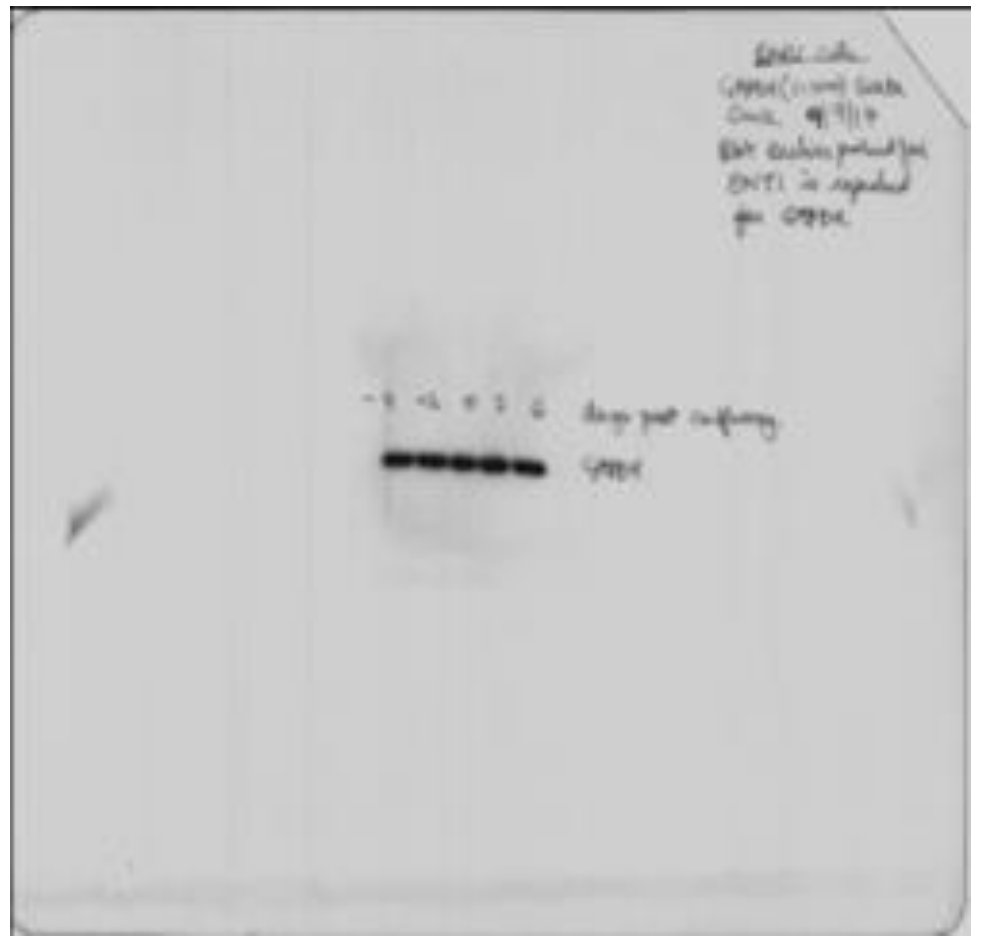

Supplement: Supplementary file 2 [file jci-136-183474-s247.pdf]
